# Three-component reactions of conjugated dienes, CH acids and formaldehyde under diffusion mixing conditions

**DOI:** 10.3762/bjoc.21.18

**Published:** 2025-02-04

**Authors:** Dmitry E Shybanov, Maxim E Kukushkin, Eugene V Babaev, Nikolai V Zyk, Elena K Beloglazkina

**Affiliations:** 1 Department of Chemistry, M. V. Lomonosov Moscow State University, 119991 Moscow, Russian Federation

**Keywords:** aldol condensation, [4 + 2]-cycloaddition, diffusion mixing, formaldehyde, Knoevenagel condensation, three-component reactions

## Abstract

A diffusion mixing technique with a volatile reagent was successfully used to generate crotonic condensation adducts of active methylene compounds and formaldehyde at room temperature in the absence of strong acids and bases. The formed adducts were highly reactive intermediates capable of reacting with dienes in a three-component reaction, leading to the formation of Diels–Alder main reaction products.

## Introduction

**Scheme 1:**
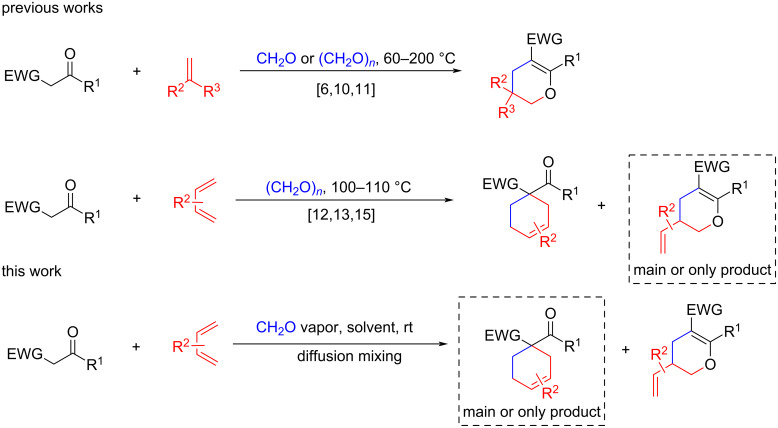
Knoevenagel and Diels–Alder reactions in the multicomponent synthesis of substituted cyclohexadienes under formaldehyde participation.

Formaldehyde is a reactive electrophilic reagent widely used as a C1 building block in multicomponent reactions [1–3]. Its role in most cases is to generate highly reactive species in situ from the nucleophilic reaction component. This can subsequently interact with other reaction components to form target products. Compared to the crotonic condensation products of other aliphatic and aromatic aldehydes, methylidene adducts of formaldehyde condensation are formed under milder conditions and are highly reactive, which is important for further synthetic transformations. However, due to the high carbonyl reactivity of formaldehyde, its interaction with active methylene compounds is often complicated by polycondensation [4–6] and polymerization processes of unstable methylidene adducts [7–9], instead of the desired formation of a monocrotonic product. Therefore, heating [6,10–14] or Lewis acid activation [15–18] have often been used in the literature for successful multicomponent reactions that proceed through the formation of formaldehyde condensation products (Scheme 1).

Previously, we have proposed a convenient diffusion mixing technique for multicomponent reactions based on the absorption of volatile reagent vapors by a mixture containing the remaining reaction components. This method was successfully used to generate highly active nitrile oxides and nitrilimines for 1,3-dipolar cycloaddition reactions [19–21]. Based on our previous experience with diffusion mixing, we assumed that formaldehyde vapor diffusion into the reaction would lead to an extremely low concentration, which should significantly reduce undesirable polycondensations involving CH_2_O as well as polymerization of the intermediately formed methylidene derivatives. In this work, we generated methylidene adducts by formaldehyde condensation under diffusion mixing conditions in three-component reactions with various CH acid derivatives and conjugated dienes (cyclopentadiene, 1,3-cyclohexadiene, 2,3-dimethylbutadiene and isoprene), leading to [4 + 2]-cycloaddition adducts (Scheme 1).

It should be noted that in previous works describing three-component reactions of carbonyl compounds, conjugated dienes and formaldehyde (generated from paraformaldehyde upon heating), an adduct of a methylidene derivative of a CH acid dienophile was detected in some cases only in reference [15] (when carrying out the reaction in a sealed tube in the presence of copper(II) acetate). However, in most reactions only the hetero-Diels–Alder products were isolated from the reaction mixtures. In the present study, we carried out similar three-component reactions under significantly milder conditions (room temperature), and the main or only [4 + 2]-cycloaddition products in most cases were carbocyclic ones.

## Results and Discussion

### Interaction of 1,3-diketone **1** with formaldehyde

**Figure 1:**
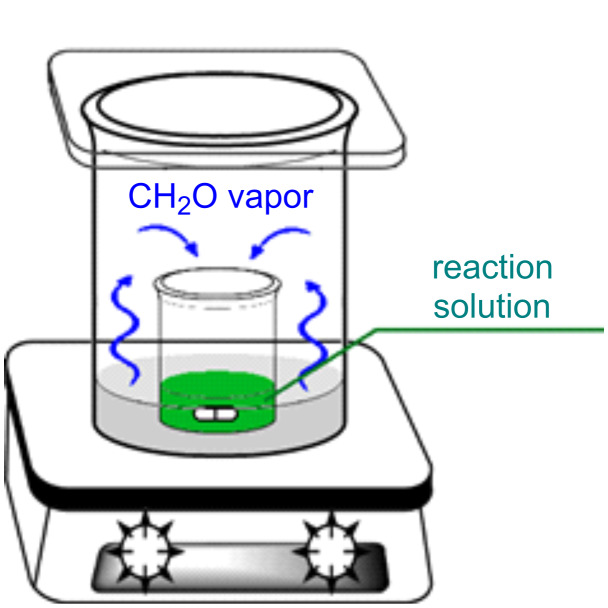
Equipment for carrying out reactions by the diffusion mixing method.

First, we investigated the ability of formaldehyde to interact with 1,3-diketone **1** using the diffusion mixing technique. For this purpose, we carried out a series of reactions in a very simple device consisting of a large vial with formalin, inside which we placed an open small vial with a solution of the other starting compounds (Figure 1). During the reaction, formaldehyde vapors from the outer vial were slowly absorbed by the mixture in the inner vial, which ensured an extremely low concentration of CH_2_O and intermediates in the reaction. An alternative to the diffusion mixing method is the slow dropwise addition of a reagent to the reaction mixture. However, in the latter case it is not possible to achieve uniform generation of active intermediates due to their formation in a local area, which accelerates unwanted side processes [19].

**Scheme 2:**
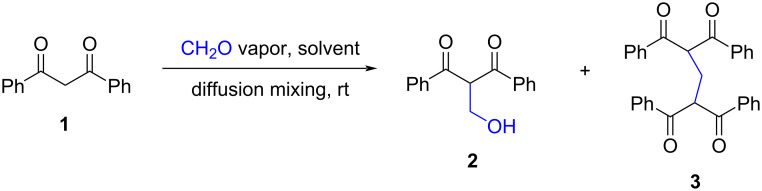
Interaction of diketone **1** with formaldehyde under the diffusion mixing conditions.

The results obtained are summarized in Scheme 2 and Table 1. It was found that the size of the vial used to carry out the reaction significantly affected the reaction rate. Thus, in an apparatus consisting of two vials with diameters of 1.3 and 2.8 cm, the conversion of the starting compound was 37 and 31%, respectively, when carrying out the reaction in chloroform or methanol over two days (Table 1, entries 3 and 7). In turn, in a system of two vials with diameters of 3.7 and 7.5 cm, and with a loading of diketone **1** that was five times greater, more than two thirds of the starting compound reacted within 24 h (Table 1, entries 11 and 13). Apparently, an increase in the surface area of the reaction solution promoted more efficient absorption of CH_2_O molecules, which accelerated the condensation reaction.

**Table 1:** Interaction of diketone **1** with formaldehyde under various conditions.

entry	conditions	conversion of **1**^a^ [%]	yield of **2**^a^ [%]	yield of **3**^a^ [%]

1	petroleum ether, **2d**^b^	57	16	30
2	petroleum ether, **7d**^b^	58	17	25
3	CHCl_3_, **2d**^b^	37	30	trace
4	CHCl_3_, **7d**^b^	77	48	21
5	CDCl_3_, **2d**^c^	65	42	0
6	Et_2_O, **2d**^b^	13	8	0
7	MeOH, **2d**^b^	31	0	17
8	EtOH, **2d**^b^	68	0	64
9	MeCN, **2d**^b^	39	30	trace
10	ʟ-proline (5 mol %), MeCN, **1d**^b^	100	0	99
11	CHCl_3_, **1d**^d^	82	48	trace
12	CHCl_3_, **2d**^d^	96	27	31
13	MeOH, **1d**^d^	69	29	trace

^a^Based on ^1^H NMR spectra of the reaction mixture. Compound **3** could be isolated in pure form, but compound **2** was unstable when isolated. ^b^Inner vial: diameter 1.3 cm, 0.09 mmol compound **1** in 5 mL solvent. Outer vial: diameter 2.8 cm, 3 mL of formalin. ^c^Inner vial: diameter 1.3 cm, 0.09 mmol compound **1** in 3 mL CDCl_3_. Outer vial: diameter 2.8 cm, 4 mL of formalin. After completion of the reaction, 550 μL of the solution were immediately placed in an ampule and analyzed by ^1^H NMR spectroscopy. ^d^Inner vial: diameter 3.7 cm, 0.45 mmol compound **1** in 10 mL solvent. Outer vial: diameter 7.5 cm, 6 mL of formalin.

The reaction of formaldehyde and diketone **1** in chloroform and acetonitrile predominantly led to diketo alcohol **2**. Its ^1^H NMR spectrum contained a triplet with a coupling constant of 5.1 Hz at 5.52 ppm and a doublet at 4.30 ppm. In methanol, ethanol and petroleum ether, with an increasing reaction time, a significant amount of product **3** was observed in the ^1^H NMR spectra of the mixtures, as identified by characteristic triplets at 5.76 and 2.77 ppm (*J* = 7.0 Hz). Compound **3** was also formed in almost quantitative yield in acetonitrile within one day in the presence of ʟ-proline (Table 1, entry 10), which is an effective catalyst for crotonic condensation [22]. Since the reaction of diketone **1** in the presence and absence of ʟ-proline was carried out under otherwise identical conditions in the same apparatus (Table 1, entries 9 and 10), the significant difference in the conversion of compound **1** could not be explained only by the evaporation and absorption processes of CH_2_O. Apparently, the reaction rate also played an important role; the presence of the condensation catalyst ʟ-proline accelerated the absorption of formaldehyde vapors by the reaction mixture. Carrying out the same reaction in the absence of a catalyst in CDCl_3_ and subsequent ^1^H NMR spectroscopy analysis showed that the mixture contained 42% of keto alcohol **2** and 35% of diketone **1** (Table 1, entry 5), and only trace amounts of CH_2_O were present. This meant that formaldehyde introduction into the reaction mixture was very limited. Therefore, the condensation rate was the limiting step of the diffusion mixing.

The rapid formation of compound **3** in a high yield in the presence of ʟ-proline could be explained by the efficient crotonic condensation of formaldehyde and acetylacetone (**1**), followed by the addition of the second equivalent of diketone **1** to the highly reactive methylidene intermediate. The mild reaction conditions (room temperature) are worth mentioning since according to the literature, unstable crotonic condensation adducts are usually generated upon heating [6,10–13,15], in acidic medium [15,23] or using oxidizing agents [24–25].

### Three-component reactions under diffusion mixing conditions

**Scheme 3:**
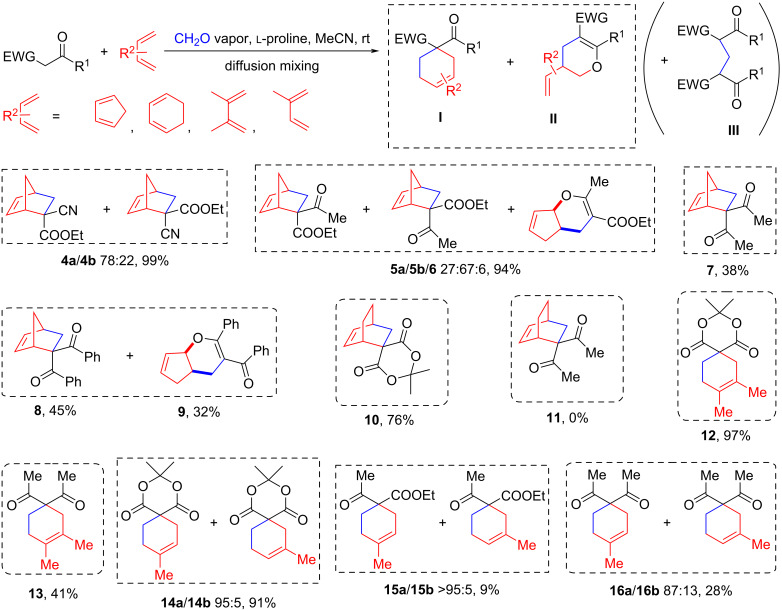
Products of three-component reactions of methylene derivatives, formaldehyde and various dienes.

Under optimized conditions, we studied the possibility of generating active methylidene derivatives from malonic ester, Meldrum's acid, cyanoacetic acid ester, acetoacetic ester, acetylacetone and 1,3-diphenylpropane-1,3-dione (**1**). We found that, with the exception of malonic ester, all compounds reacted with formaldehyde under the above conditions to form highly reactive intermediates capable of [4 + 2]-cycloaddition reactions. Cyclopentadiene, cyclohexadiene, 2,3-dimethylbutadiene and isoprene were used as traps for the intermediates formed. The results are presented in Scheme 3. In general, the reactions produced adducts of the Diels–Alder (i.e., **I**) and the hetero-Diels–Alder reaction (i.e., **II**), or adducts resulting from the addition of a second equivalent of CH acid to the crotonic condensation product (i.e., **III**).

**Scheme 4:**
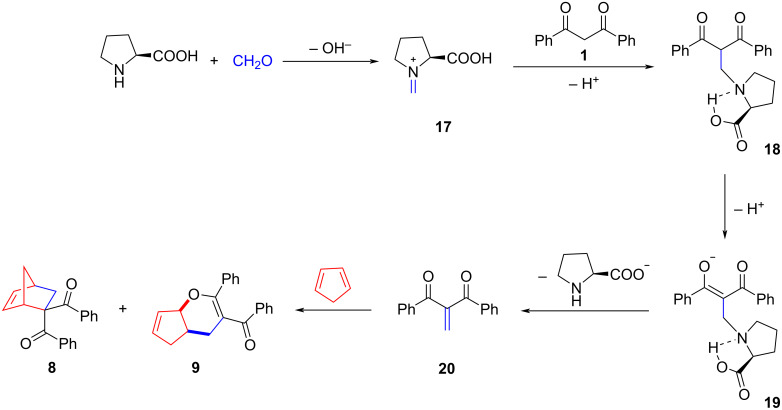
Proposed mechanism for the formation of compounds **8** and **9** in the presence of ʟ-proline.

Apparently ʟ-proline played an essential role as catalyst in this three-component reaction. Using compounds **8** and **9**, we could show that when the reaction was carried out in the absence of ʟ-proline, the conversion of the starting CH acid **1** after 5 days was less than 50%, and the main products present in the mixture were compounds **2** and **3**. The proposed mechanism for the formation of compounds **8** and **9** with ʟ-proline participation is shown in Scheme 4. In the first step, formaldehyde reacts with proline, forming an imine salt **17**, which then reacts with the diketone **1**. The resulting intermediate **18** eliminates a proton and the anion of ʟ-proline, and then the methylenebenzophenone **20** reacts with cyclopentadiene to form the final products **8** and **9**.

When using cyclopentadiene, it turned out that byproducts **III** were practically not formed, and the target compounds **4**–**6**, **8** and **9** were isolated in a high overall yield. The moderate yield of compound **7** was due to the partial evaporation of the starting acetylacetone from the inner vial into the outer vessel containing formaldehyde, which affected the yield of all [4 + 2]-cycloaddition adducts involving this CH acid, regardless of the choice of diene. For acetoacetic ester and 1,3-diphenylpropane-1,3-dione, it was found that in addition to the main products **5** and **8**, the reaction also produced hetero-Diels–Alder reaction adducts **6** and **9**. For these, CH–O ^1^H NMR signals in the region of 4.9–5.4 ppm were characteristic.

**Scheme 5:**
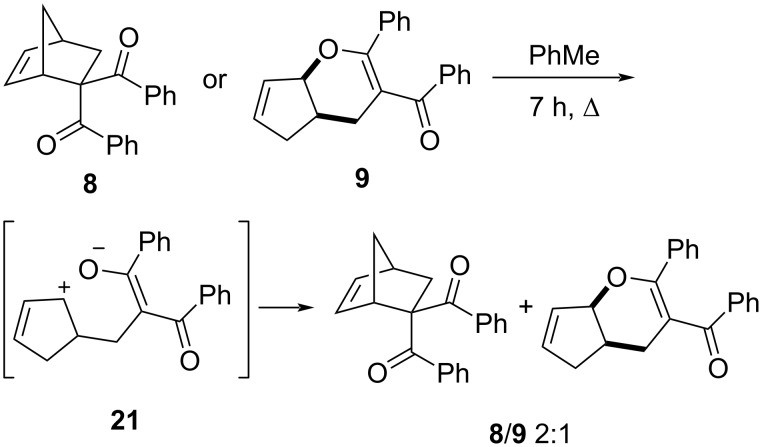
Interconversion of derivatives **8** and **9**.

It is worth noting that the individual isolated compounds **8** and particularly **9** were unstable when stored in solution, and boiling adduct **8** or **9** in toluene for 7 h led to the formation of an equilibrium mixture of these compounds in a ratio of ≈2:1 (Scheme 5).

We propose that the reversible transformation of **8** to **9** proceeded via the intermediate formation of zwitterion **21**, in which the charges were stabilized by mesomeric effects under participation of the C=C bond. Apparently, the ratio of the adducts of the Diels–Alder (i.e., **I**) and the hetero-Diels–Alder reaction (i.e., **II**) was strongly influenced by steric factors; a decreased steric hindrance in the initial CH acid derivatives led to a more selective formation of the structures **I**. This was confirmed through the three-component reaction with acetylacetone, in which only the product **7** was formed (according to ^1^H NMR spectra of the reaction mixture).

**Scheme 6:**
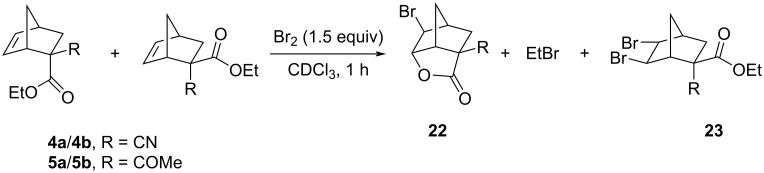
Interaction of **4a**/**4b** and **5a**/**5b** mixtures with bromine.

Stereoselective interactions between methylidene adducts and cyclopentadiene were confirmed for three-component reactions involving cyanoacetic ester and acetylacetone. The configuration of the products **4** and **5** was established by bromination of a small amount of these isomeric compounds in CDCl_3_ (Scheme 6) and subsequent analysis of the mixture by ^1^H NMR spectroscopy. For the stereoisomers **4**, the main product was lactone **22**, identified by the signal of the CH–O group at 5.03 ppm, as well as by ethyl bromide, which indicated the predominance of isomer **4a** in the mixture. For isomers **5**, preferential formation of dibromide **23** and products of carbocationic rearrangements containing an ester group, the precursor of which was compound **5b**, were found. The preferential formation of diastereomers **4a** and **5b** during the Diels–Alder reaction with the corresponding methylidene adducts was consistent with literature data [24,26].

Next, we studied three-component condensation reactions in the presence of the less active dienes cyclohexadiene, 2,3-dimethylbutadiene and isoprene. In reactions with Meldrum's acid, target products **10**, **12** and **14** were obtained in a high yield. But for other CH acid derivatives, the reactions proceeded much less successful due to the side formation of products **III**. Under general conditions, the least active cyclohexadiene did not react even with acetylacetone, and for 2,3-dimethylbutadiene, target compound **13** was obtained in 41% yield. In all cases, no formation of even trace amounts of the hetero-Diels–Alder reaction adducts **II** was detected.

The regioselectivity of the cycloaddition of methylidene intermediates was also studied using isoprene as an example. In reactions involving Meldrum's acid, the formation of an inseparable mixture of the two stereoisomeric products **14a** and **14b** in a ratio of 95:5 was detected with a yield of 91%, the ^1^H NMR spectra of which coincided with those described in the literature [27]. The three-component reaction with acetoacetic ester was highly selective, and the formation of a minor [4 + 2]-cycloaddition product was not observed (according to ^1^H NMR spectroscopy), but the yield of the products **15** did not exceed 9%. For acetylacetone, the reaction was the least selective, resulting in an inseparable mixture of compounds **16a** and **16b** in a ratio of 87:13 and a total yield of 28%.

## Conclusion

This work demonstrates that the technique of diffusion mixing with a volatile reagent can be successfully used to generate croton condensation adducts of active methylene compounds with formaldehyde at room temperature in the absence of strong acids and bases. These adducts are highly reactive intermediates capable of reacting with dienes in three-component reactions, leading to the formation of Diels–Alder main products or hetero-Diels–Alder adducts. In some cases, Michael addition products due to the addition of another equivalent of active methylene compound were also observed. Among the reactions studied, the diffusion mixing method gave the highest yield when using Meldrum’s acid as CH acid and in reactions with cyclopentadiene as diene.

## Experimental

### Materials and methods

All solvents used were purified and dehydrated using the methods described in reference [28]. All starting reagents were purchased from commercial sources (e.g., Sigma-Aldrich, abcr, AKSci). Reactions were checked by TLC analysis using silica plates with a fluorescent indicator (254 nm) and visualized with a UV lamp. ^1^H and ^13^C NMR spectra were recorded on Bruker Avance and Agilent 400-MR spectrometers (400 MHz for ^1^H, 100 MHz for ^13^C). Chemical shifts are reported in ppm relative to TMS.

### General procedure for the three-component reactions under diffusion mixing conditions

A mixture of 1.0 mmol CH acid (1 equiv), 0.05 mmol ʟ-proline and 2.0–5.0 mmol diene (2–5 equiv) in 3–5 mL acetonitrile was placed into a 15 mL vial (diameter 1.3 cm). This vial was then placed into a closed 50 mL vial (diameter 2.8 cm) containing 3–5 mL of formalin, and the reaction mixture was stirred at room temperature for 2 days (TLC or NMR control). After completion of the reaction, the solvent was removed under reduced pressure, and the residue was purified by column chromatography on silica gel using chloroform as eluent.

((1*S*,4*S*)-Bicyclo[2.2.1]hept-5-ene-2,2-diyl)bis(phenylmethanone) (**8**) and phenyl((4a*R*,7a*S*)-2-phenyl-4,4a,5,7a-tetrahydrocyclopenta[*b*]pyran-3-yl)methanone (**9**). From 1,3-diphenylpropane-1,3-dione (224 mg, 1.0 mmol), ʟ-proline (6 mg, 0.05 mmol) and cyclopentadiene (330 mg, 5.0 mmol), compounds **8** (136 mg, 45%) and **9** (97 mg, 32%) were obtained as white crystalline solids.

### Major isomer **8**

^1^H NMR (400 MHz, CDCl_3_, δ) 7.98–7.89 (m, 4H), 7.45–7.38 (m, 2H), 7.36–7.28 (m, 4H), 6.30 (dd, *J* = 5.7, 3.0 Hz, 1H), 5.74 (dd, *J* = 5.7, 2.9 Hz, 1H), 3.94–3.90 (m, 1H), 3.00–2.95 (m, 1H), 2.84 (dd, *J* = 12.1, 2.9 Hz, 1H), 2.18 (dd, *J* = 12.1, 3.7 Hz, 1H), 1.76–1.71 (m, 1H), 1.63–1.54 (m, 1H); ^13^C NMR (101 MHz, CDCl_3_, δ) 200.0, 197.0, 140.1, 137.5, 136.6, 133.1, 133.0, 132.7, 129.9 (2C), 129.2 (2C), 128.6 (2C), 128.5 (2C), 71.9, 51.6, 49.3, 43.0, 36.9; HRMS–ESI^+^ (*m*/*z*): [M + H]^+^ calcd for C_21_H_19_O_2_, 303.1380; found, 303.1382.

### Minor isomer **9**

^1^H NMR (400 MHz, CDCl_3_, δ) 7.55–7.49 (m, 2H), 7.22–7.15 (m, 3H), 7.11–6.99 (m, 5H), 6.22–6.16 (m, 1H), 6.09–6.04 (m, 1H), 5.44–5.39 (m, 1H), 3.18–3.08 (m, 1H), 2.75 (dd, *J* = 14.3, 6.1 Hz, 1H), 2.71–2.62 (m, 1H), 2.58 (dd, *J* = 14.3, 4.8 Hz, 1H), 2.34–2.25 (m, 1H); ^13^C NMR (101 MHz, CDCl_3_, δ) 198.4, 165.1, 139.1, 137.5, 135.6, 131.4, 130.9, 129.7 (2C), 129.6 (2C), 129.5 (2C), 127.7 (3C), 115.0, 85.6, 39.3, 37.8, 27.4; HRMS–ESI^+^ (*m*/*z*): [M + H]^+^ calcd for C_21_H_19_O_2_, 303.1380; found, 303.1383.

## Supporting Information

File 1 ^1^H and ^13^C NMR spectra of synthesized compounds.

## Data Availability

All data that supports the findings of this study is available in the published article and/or the supporting information of this article.
