# Supplementary material for: Three-component reactions of conjugated dienes, CH acids and formaldehyde under diffusion mixing conditions
Source: Beilstein J Org Chem. 2025 Feb 4;21:262–9. doi: 10.3762/bjoc.21.18 (PMC11809574; doi:10.3762/bjoc.21.18)
Supplement: File 1 — 1H and 13C NMR spectra of synthesized compounds. [file Beilstein_J_Org_Chem-21-262-s001.pdf]

BEILSTEIN JOURNAL OF ORGANIC CHEMISTRY

Supporting Information

for

Three-component reactions of conjugated dienes, CH acids and formaldehyde under diffusion mixing conditions

Dmitry E. Shybanov, Maxim E. Kukushkin, Eugene V. Babaev, Nikolai V. Zyk and Elena K. Beloglazkina

Beilstein J. Org. Chem. **2025,** *21,* 262–269. doi:10.3762/bjoc.21.18

^{1}H and ^{13}C NMR spectra of synthesized compounds

License and Terms: This is a supporting information file under the terms of the Creative Commons Attribution License (https://creativecommons.org/licenses/by/4.0). Please note that the reuse, redistribution and reproduction in particular requires that the author(s) and source are credited and that individual graphics may be subject to special legal provisions.
The license is subject to the *Beilstein Journal of Organic Chemistry* terms and conditions: (https://www.beilstein-journals.org/bjoc/terms)

Table of contents

1. Synthesis S2

2. ^{1}H and ^{13}C NMR spectra of the compounds **3**–**10** and **12**–**16** S6

3. ^{1}H NMR spectra of reaction mixtures formed during bromination of compounds **4** and **5** S18

4. LC–MS/MS spectra of compound mixtures S20

References S23

1. Synthesis

2,4-dibenzoyl-1,5-diphenylpentane-1,5-dione (**3**) [1]. From 1,3-diphenylpropane-1,3-dione (112 mg, 0.50 mmol) and L-proline (3 mg, 0.025 mmol) compound **3** (227 mg, 99%) was obtained as a white crystalline solid.

^{1}H NMR (400 MHz, $CDCl_3$): δ 8.20-8.12 (m, 8H), 7.63-7.57 (m, 4H), 7.54-7.45 (m, 8H), 5.76 (t, *J* = 7.0 Hz, 2H), 2.77 (t, *J* = 7.0 Hz, 2H). **^{13}C NMR** (101 MHz, $CDCl_3$): δ 196.7 (4C), 135.6 (4C), 134.0 (4C), 129.2 (8C), 128.9 (8C), 54.1 (2C), 29.1.

HRMS (ESI+) m/z calcd. for ($C_{31}H_{25}O_4$, M+H): 461.1747, found: (M+H): 461.1753.

Ethyl (1S,2S*,4S*)-2-cyanobicyclo[2.2.1]hept-5-ene-2-carboxylate* (**4a**) and *ethyl (1S*,2R*,4S*)-2-cyanobicyclo[2.2.1]hept-5-ene-2-carboxylate* (**4b**) [2]. From cyanoacetic acid ester (113 mg, 1.0 mmol), L-proline (6 mg, 0.05 mmol) and cyclopentadiene (330 mg, 5.0 mmol) the mixture of compounds **4a** and **4b** in 78/22 ratio (189 mg, 99%) was obtained as a light orange oil.

Major isomer 4a: ^{1}H NMR (400 MHz, $CDCl_3$): δ 6.31 (dd, *J* = 5.5, 2.9 Hz, 1H), 5.86 (dd, *J* = 5.5, 2.9 Hz, 1H), 4.19 (qd, *J* = 7.2, 3.2 Hz, 2H), 3.56-3.51 (m, 1H), 3.10-3.06 (m, 1H), 2.23 (dd, *J* = 12.5, 3.7 Hz, 1H), 2.05 (dd, *J* = 12.5, 2.9 Hz, 1H), 1.79-1.73 (m, 1H), 1.67-1.59 (m, 1H), 1.29 (t, *J* = 7.2 Hz, 3H). **^{13}C NMR** (101 MHz, $CDCl_3$): δ 167.4, 140.3, 131.0, 121.7, 62.7, 53.6, 49.2 (2C), 42.9, 37.2, 14.1.

Minor isomer 4b: ^{1}H NMR (400 MHz, $CDCl_3$): δ 6.43 (dd, *J* = 5.8, 3.0 Hz, 1H), 6.33-6.27 (m, 1H), 4.27 (q, *J* = 7.1 Hz, 2H), 3.38–3.35 (m, 1H), 3.10-3.06 (m, 1H), 2.54 (dd, *J* = 12.4, 3.4 Hz, 1H), 1.69 (dd, *J* = 12.4, 2.7 Hz, 1H), 1.67–1.59 (m, 1H), 1.54-1.49 (m, 1H), 1.33 (t, *J* = 7.1 Hz, 3H). **^{13}C NMR** (101 MHz, $CDCl_3$): δ 167.4, 141.0, 134.1, 121.1, 63.0, 52.4, 46.8, 46.5, 42.7, 39.1, 14.1.

HRMS (ESI+) m/z calcd. for ($C_{11}H_{14}NO_2$, M+H): 192.1019, found: (M+H): 192.1020.

Ethyl (1S,2S*,4S*)-2-acetylbicyclo[2.2.1]hept-5-ene-2-carboxylate* (**5a**), *ethyl (1S*,2R*,4S*)-2-acetylbicyclo[2.2.1]hept-5-ene-2-carboxylate* (**5b**) and *ethyl (4aR*,7aS*)-2-methyl-4,4a,5,7a-tetrahydrocyclopenta[b]pyran-3-carboxylate* (**6**). From acetoacetic ester (130 mg, 1.0 mmol), L-proline (6 mg, 0.05 mmol) and cyclopentadiene (330 mg, 5.0 mmol) the mixture of compounds **5a**, **5b** and **6** in 27/67/6 ratio (196 mg, 94%) was obtained as a colorless oil.

Minor isomer 5a: ^{1}H NMR (400 MHz, $CDCl_3$): δ 6.23 (dd, *J* = 5.6, 3.0 Hz, 1H), 5.96 (dd, *J* = 5.6, 2.9 Hz, 1H), 4.20-4.05 (m, 2H), 3.33 (s, 1H), 2.85 (s, 1H), 2.15 (s, 3H), 2.05-1.97 (m, 2H), 1.45-1.41 (m, 1H), 1.38-1.33 (m, 1H), 1.24-1.17 (m, 3H). **^{13}C NMR** (101 MHz, $CDCl_3$): δ 204.4, 171.7, 140.0, 133.9, 67.9, 61.3, 48.4, 48.3, 42.2, 34.4, 27.2, 14.1.

Major isomer 5b: ^{1}H NMR (400 MHz, $CDCl_3$): δ 6.17 (dd, *J* = 5.6, 3.1 Hz, 1H), 5.88 (dd, *J* = 5.6, 2.8 Hz, 1H), 4.20-4.05 (m, 2H), 3.41 (s, 1H), 2.85 (s, 1H), 2.08 (s, 3H), 2.02-1.97 (m, 1H), 1.95-1.92 (m,

1H), 1.62-1.58 (m, 1H), 1.51-1.46 (m, 1H), 1.24-1.17 (m, 3H). **^{13}C NMR** (101 MHz, $CDCl_3$): δ 202.5, 172.9, 140.1, 132.3, 67.6, 61.6, 49.5, 49.1, 42.2, 33.9, 27.5, 14.1.

Minor isomer 6: ^{1}H NMR (400 MHz, $CDCl_3$) *(due to the low content of compound **6** in the mixture and the overlap of most signals with the signals of the main isomer, only characteristic peaks are indicated):* δ 5.96-5.93 (m, 1H), 5.82-5.77 (m, 1H), 4.93 (d, *J* = 6.9 Hz, 1H).

HRMS (ESI+) m/z calcd. for ($C_{12}H_{17}O_3$, M+H): 209.1172, found: (M+H): 209.1175.

1,1'-(Bicyclo[2.2.1]hept-5-ene-2,2-diyl)bis(ethan-1-one) (**7**). From acetylacetone (100 mg, 1.0 mmol), L-proline (6 mg, 0.05 mmol) and cyclopentadiene (330 mg, 5.0 mmol) compound **7** (63 mg, 38%) was obtained as a colorless oil.

^{1}H NMR (400 MHz, $CDCl_3$): δ 6.17 (dd, *J* = 5.7, 3.1 Hz, 1H), 5.92 (dd, *J* = 5.7, 2.9 Hz, 1H), 3.52-3.46 (m, 1H), 2.86-2.79 (m, 1H), 2.09-2.03 (m, 1H), 2.07 (s, 3H), 2.01 (s, 3H), 1.87 (dd, *J* = 12.2, 3.8 Hz, 1H), 1.48-1.42 (m, 1H), 1.27 (d, *J* = 8.8 Hz, 1H). **^{13}C NMR** (101 MHz, $CDCl_3$): δ 206.0, 204.5, 140.3, 132.8, 77.4, 48.8, 47.7, 42.3, 33.1, 27.8, 27.6.

HRMS (ESI+) m/z calcd. for ($C_{11}H_{15}O_2$, M+H): 179.1067, found: (M+H): 179.1065.

((1S,4S*)-Bicyclo[2.2.1]hept-5-ene-2,2-diyl)bis(phenylmethanone)* (**8**) and *phenyl((4aR*,7aS*)-2-phenyl-4,4a,5,7a-tetrahydrocyclopenta[b]pyran-3-yl)methanone* (**9**) [3].From 1,3-diphenylpropane-1,3-dione (224 mg, 1.0 mmol), L-proline (6 mg, 0.05 mmol) and cyclopentadiene (330 mg, 5.0 mmol) compound **8** (136 mg, 45%) and compound **9** (97 mg, 32%) were obtained as a white crystalline solids.

Major isomer 8: ^{1}H NMR (400 MHz, $CDCl_3$): δ 7.98-7.89 (m, 4H), 7.45-7.38 (m, 2H), 7.36-7.28 (m, 4H), 6.30 (dd, *J* = 5.7, 3.0 Hz, 1H), 5.74 (dd, *J* = 5.7, 2.9 Hz, 1H), 3.94-3.90 (m, 1H), 3.00-2.95 (m, 1H), 2.84 (dd, *J* = 12.1, 2.9 Hz, 1H), 2.18 (dd, *J* = 12.1, 3.7 Hz, 1H), 1.76-1.71 (m, 1H), 1.63-1.54 (m, 1H). **^{13}C NMR** (101 MHz, $CDCl_3$): δ 200.0, 197.0, 140.1, 137.5, 136.6, 133.1, 133.0, 132.7, 129.9 (2C), 129.2 (2C), 128.6 (2C), 128.5 (2C), 71.9, 51.6, 49.3, 43.0, 36.9.

HRMS (ESI+) m/z calcd. for ($C_{21}H_{19}O_2$, M+H): 303.1380, found: (M+H): 303.1382.

Minor isomer 9: ^{1}H NMR (400 MHz, $CDCl_3$): δ 7.55-7.49 (m, 2H), 7.22-7.15 (m, 3H), 7.11-6.99 (m, 5H), 6.22-6.16 (m, 1H), 6.09-6.04 (m, 1H), 5.44-5.39 (m, 1H), 3.18-3.08 (m, 1H), 2.75 (dd, *J* = 14.3, 6.1 Hz, 1H), 2.71-2.62 (m, 1H), 2.58 (dd, *J* = 14.3, 4.8 Hz, 1H), 2.34-2.25 (m, 1H). **^{13}C NMR** (101 MHz, $CDCl_3$): δ 198.4, 165.1, 139.1, 137.5, 135.6, 131.4, 130.9, 129.7 (2C), 129.6 (2C), 129.5 (2C), 127.7 (3C), 115.0, 85.6, 39.3, 37.8, 27.4.

HRMS (ESI+) m/z calcd. for ($C_{21}H_{19}O_2$, M+H): 303.1380, found: (M+H): 303.1383.

(1S,4S*)-2',2'-dimethylspiro[bicyclo[2.2.2]octane-2,5'-[1,3]dioxan]-5-ene-4',6'-dione* (**10**) [4]. From Meldrum's acid (144 mg, 1.0 mmol), L-proline (6 mg, 0.05 mmol) and 1,3-cyclohexadiene (160 mg, 2.0 mmol) compound **10** (179 mg, 76%) was obtained as a white crystalline solid.

^{1}H NMR (400 MHz, $CDCl_3$): δ 6.45-6.39 (m, 1H), 6.10-6.04 (m, 1H), 2.99-2.93 (m, 1H), 2.81-2.73 (m, 1H), 2.17 (dd, J = 12.8, 2.7 Hz, 1H), 2.02-1.93 (m, 1H), 1.78 (s, 3H), 1.76-1.70 (m, 2H), 1.63 (s, 3H), 1.28-1.10 (m, 2H). **^{13}C NMR** (101 MHz, $CDCl_3$): δ 169.0, 168.9, 136.3, 128.9, 105.2, 54.5, 39.7, 32.5, 30.5, 29.2, 28.1, 22.3, 22.1.

HRMS (ESI+) m/z calcd. for ($C_{13}H_{17}O_4$, M+H): 237.1121, found: (M+H): 237.1123.

3,3,8,9-Tetramethyl-2,4-dioxaspiro[5.5]undec-8-ene-1,5-dione (**12**) [5]. From Meldrum's acid (144 mg, 1.0 mmol), L-proline (6 mg, 0.05 mmol) and 2,3-dimethyl-1,3-butadiene (164 mg, 2.0 mmol) compound **12** (231 mg, 97%) was obtained as a white crystalline solid.

^{1}H NMR (400 MHz, $CDCl_3$): δ 2.49-2.48 (m, 2H), 2.12-2.11 (m, 4H), 1.75 (s, 3H), 1.72 (s, 3H), 1.68 (s, 3H), 1.65 (s, 3H). **^{13}C NMR** (101 MHz, $CDCl_3$): δ 169.9 (2C), 124.9, 121.1, 104.8, 48.6, 36.7, 31.4, 29.7, 28.6, 28.0, 19.1, 18.7.

HRMS (ESI+) m/z calcd. for ($C_{13}H_{19}O_4$, M+H): 239.1278, found: (M+H): 239.1270.

1,1'-(3,4-dimethylcyclohex-3-ene-1,1-diyl)bis(ethan-1-one) (**13**). From acetylacetone (100 mg, 1.0 mmol), L-proline (6 mg, 0.05 mmol) and 2,3-dimethyl-1,3-butadiene (164 mg, 2.0 mmol) compound **13** (80 mg, 41%) was obtained as a colorless oil.

^{1}H NMR (400 MHz, $CDCl_3$): δ 2.34 (bs, 2H), 2.04 (s, 6H), 2.04-2.01 (m, 2H), 1.92-1.85 (m, 2H), 1.61 (s, 3H), 1.50 (s, 3H). **^{13}C NMR** (101 MHz, $CDCl_3$): δ 206.7 (2C), 125.4, 122.8, 67.1, 35.1, 28.8, 27.3, 26.1 (2C), 19.2, 18.7.

HRMS (ESI+) m/z calcd. for ($C_{12}H_{19}O_2$, M+H): 195.1380, found: (M+H): 195.1381.

3,3,9-Trimethyl-2,4-dioxaspiro[5.5]undec-8-ene-1,5-dione (**14a**) and *3,3,8-trimethyl-2,4-dioxaspiro[5.5]undec-8-ene-1,5-dione* (**14b**) [6]. From Meldrum's acid (144 mg, 1.0 mmol), L-proline (6 mg, 0.05 mmol) and isoprene (340 mg, 5.0 mmol) the mixture of compounds **14a** and **14b** in 95/5 ratio (204 mg, 91%) was obtained as a white crystalline solid.

Major isomer 14a: ^{1}H NMR (400 MHz, $CDCl_3$): δ 5.38-5.33 (m, 1H), 2.59-2.55 (m, 2H), 2.17-2.12 (m, 2H), 2.11-2.06 (m, 2H), 1.74-1.71 (m, 3H), 1.70-1.66 (m, 6H). **^{13}C NMR** (101 MHz, $CDCl_3$): δ 169.8 (2C), 133.1, 116.1, 104.7, 46.9, 31.5, 30.9, 29.5, 28.5, 26.4, 23.4.

Minor isomer 14b: ^{1}H NMR (400 MHz, $CDCl_3$): δ 5.53-5.51 (m, 1H), 2.46-2.44 (m, 2H), 2.17-2.12 (m, 2H), 2.11-2.06 (m, 2H), 1.74-1.71 (m, 3H), 1.70-1.66 (m, 6H).

HRMS (ESI+) m/z calcd. for ($C_{12}H_{17}O_4$, M+H): 225.1121, found: (M+H): 225.1114.

Ethyl 1-acetyl-4-methylcyclohex-3-ene-1-carboxylate (**15a**) and *ethyl 1-acetyl-3-methylcyclohex-3-ene-1-carboxylate* (**15b**). From acetoacetic ester (130 mg, 1.0 mmol), L-proline (6 mg, 0.05 mmol) and isoprene (340 mg, 5.0 mmol) the mixture of compounds **15a** and **15b** >95/5 ratio (19 mg, 9%) was obtained as a colorless oil.

Major isomer 15a: ^{1}**H NMR** (400 MHz, $CDCl_3$): δ 5.40-5.31 (m, 1H), 4.22-4.13 (m, 2H), 2.58-2.47 (m, 1H), 2.46-2.36 (m, 1H), 2.17 (s, 3H), 2.16-2.10 (m, 1H), 2.08-1.89 (m, 3H), 1.61 (s, 3H), 1.24 (d, *J* = 7.1 Hz, 3H). ^{13}**C NMR** (101 MHz, $CDCl_3$): δ 205.4, 172.3, 133.7, 118.1, 61.5, 59.0, 30.1, 27.3, 27.2, 25.9, 23.4, 14.2.

HRMS (ESI+) m/z calcd. for ($C_{12}H_{19}O_3$, M+H): 211.1329, found: (M+H): 211.1337.

1,1'-(4-Methylcyclohex-3-ene-1,1-diyl)bis(ethan-1-one) (**16a**) and *1,1'-(3-methylcyclohex-3-ene-1,1-diyl)bis(ethan-1-one)* (**16b**). From acetylacetone (100 mg, 1.0 mmol), L-proline (6 mg, 0.05 mmol) and isoprene (340 mg, 5.0 mmol) the mixture of compounds **16a** and **16b** in 87/13 ratio (50 mg, 28%) was obtained as a light yellow oil.

Major isomer 16a: ^{1}**H NMR** (400 MHz, $CDCl_3$): δ 5.38-5.33 (m, 1H), 2.48-2.44 (m, 2H), 2.14-2.07 (m, 8H), 1.96-1.90 (m, 2H), 1.59 (s, 3H). ^{13}**C NMR** (101 MHz, $CDCl_3$): δ 206.8 (2C), 134.1, 118.1, 66.7, 29.3, 27.3, 27.1, 26.2 (2C), 23.3.

Minor isomer 16b: ^{1}**H NMR** (400 MHz, $CDCl_3$): δ 5.35-5.30 (m, 1H), 2.37-2.35 (m, 2H), 2.14-2.07 (m, 8H), 1.96-1.90 (m, 2H), 1.70 (s, 3H). ^{13}**C NMR** (101 MHz, $CDCl_3$): δ 206.6 (2C), 131.3, 120.7, 67.2, 33.7, 29.3, 26.5 (2C), 23.7, 22.6.

HRMS (ESI+) m/z calcd. for ($C_{11}H_{17}O_2$, M+H): 181.1223, found: (M+H): 181.1222.

2. ^{1}H and ^{13}C NMR spectra of the compounds 3–10 and 12–16

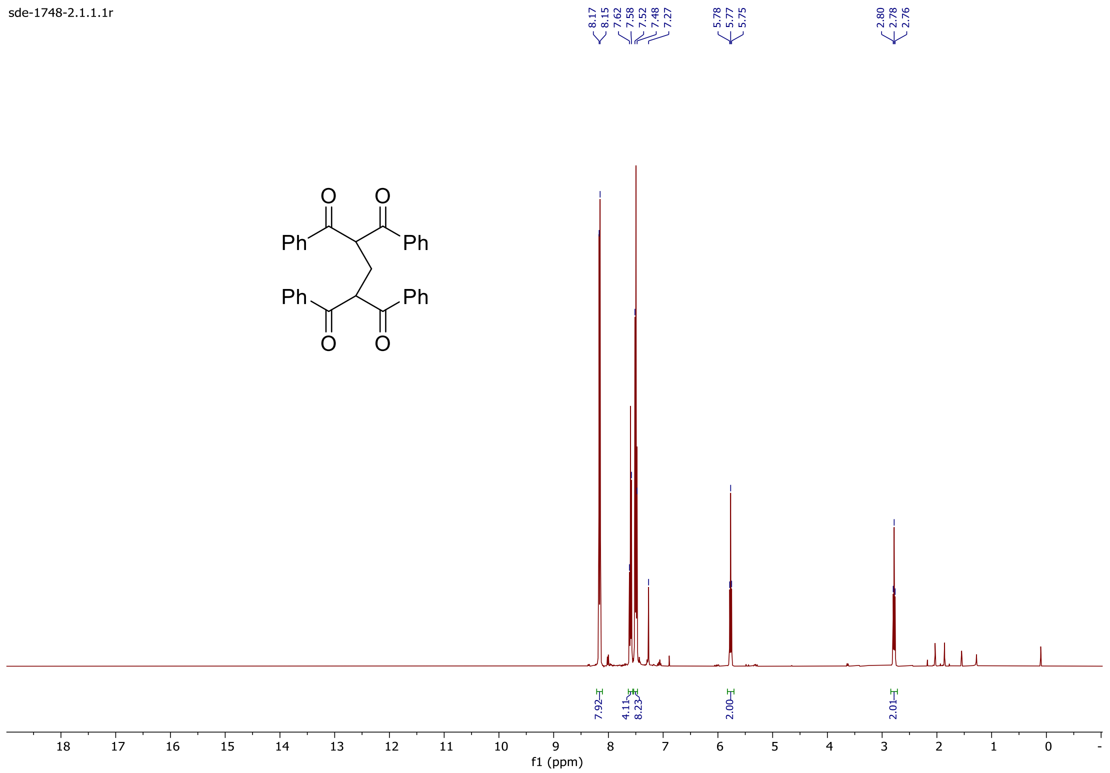

Figure S1. ^{1}H NMR spectra of compound **3.**

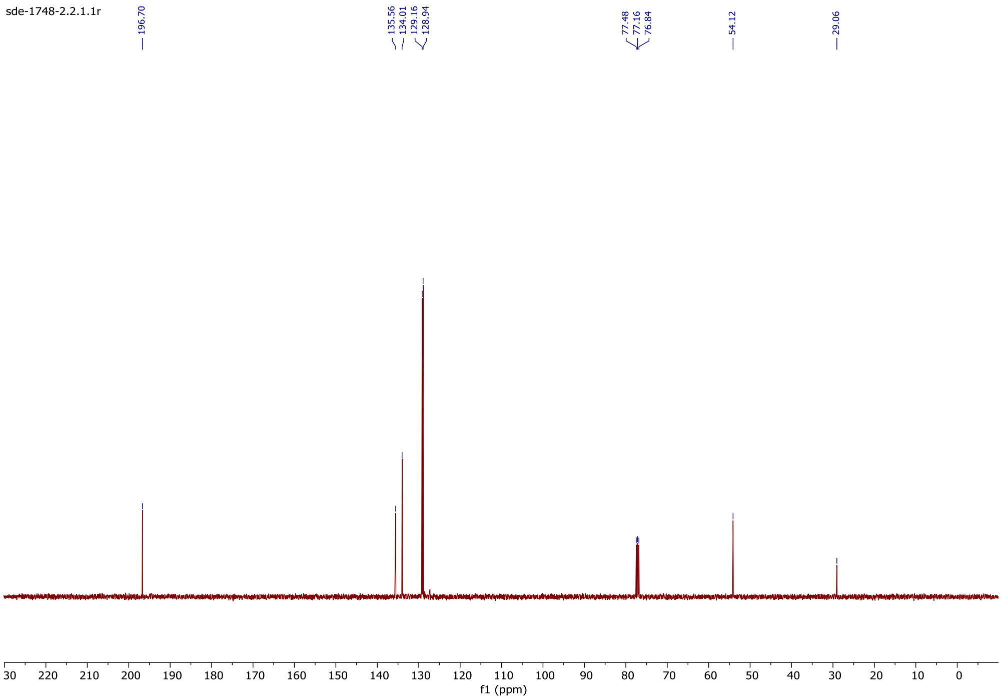

Figure S2. ^{13}C NMR spectra of compound **3.**

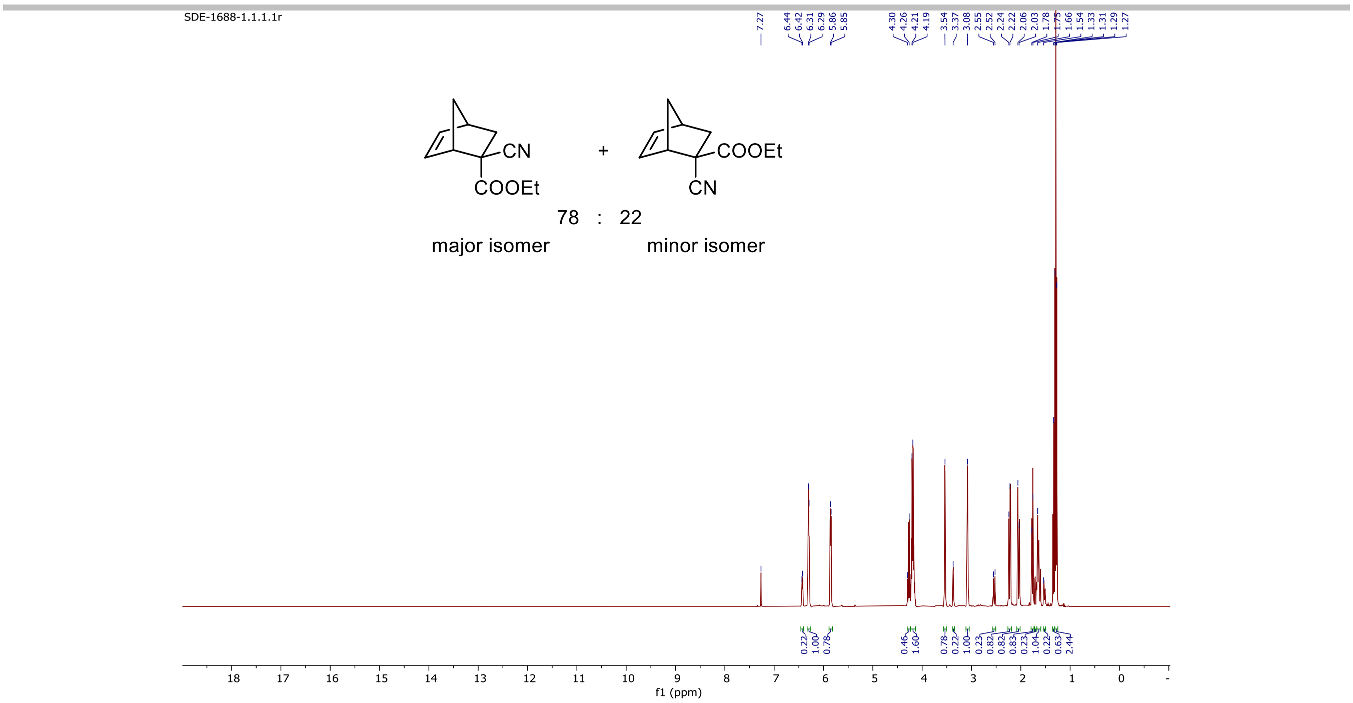

Figure S3. ^{1}H NMR spectra of the mixture of compounds **4a** and **4b.**

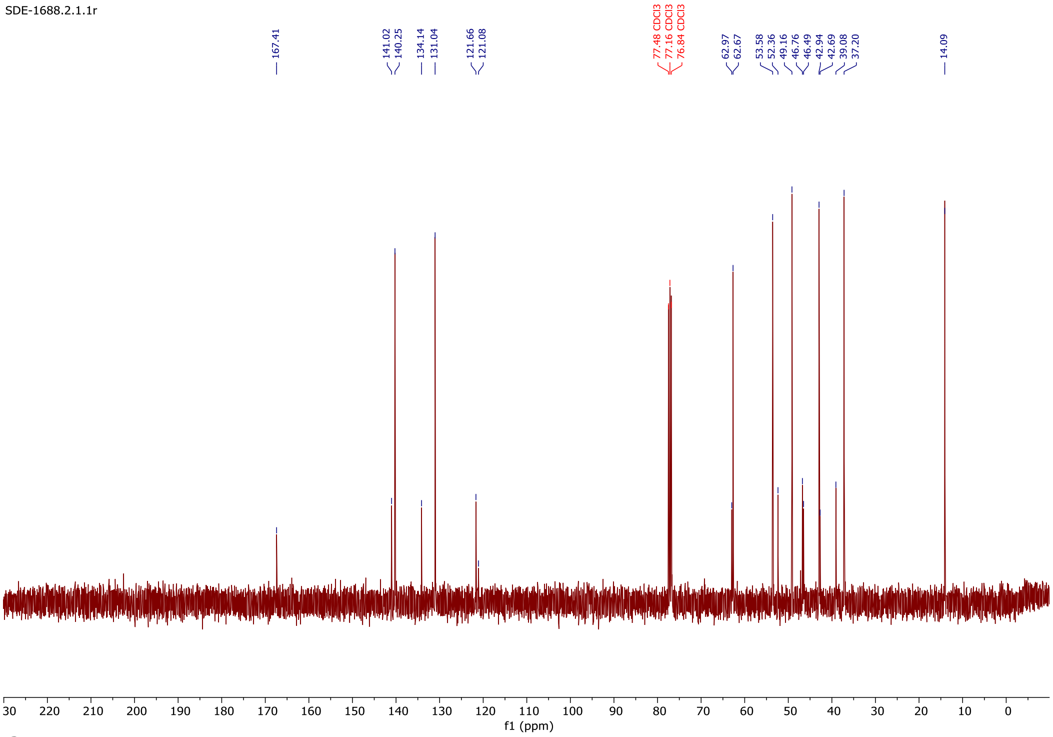

Figure S4. ^{13}C NMR spectra of the mixture of compounds **4a** and **4b.**

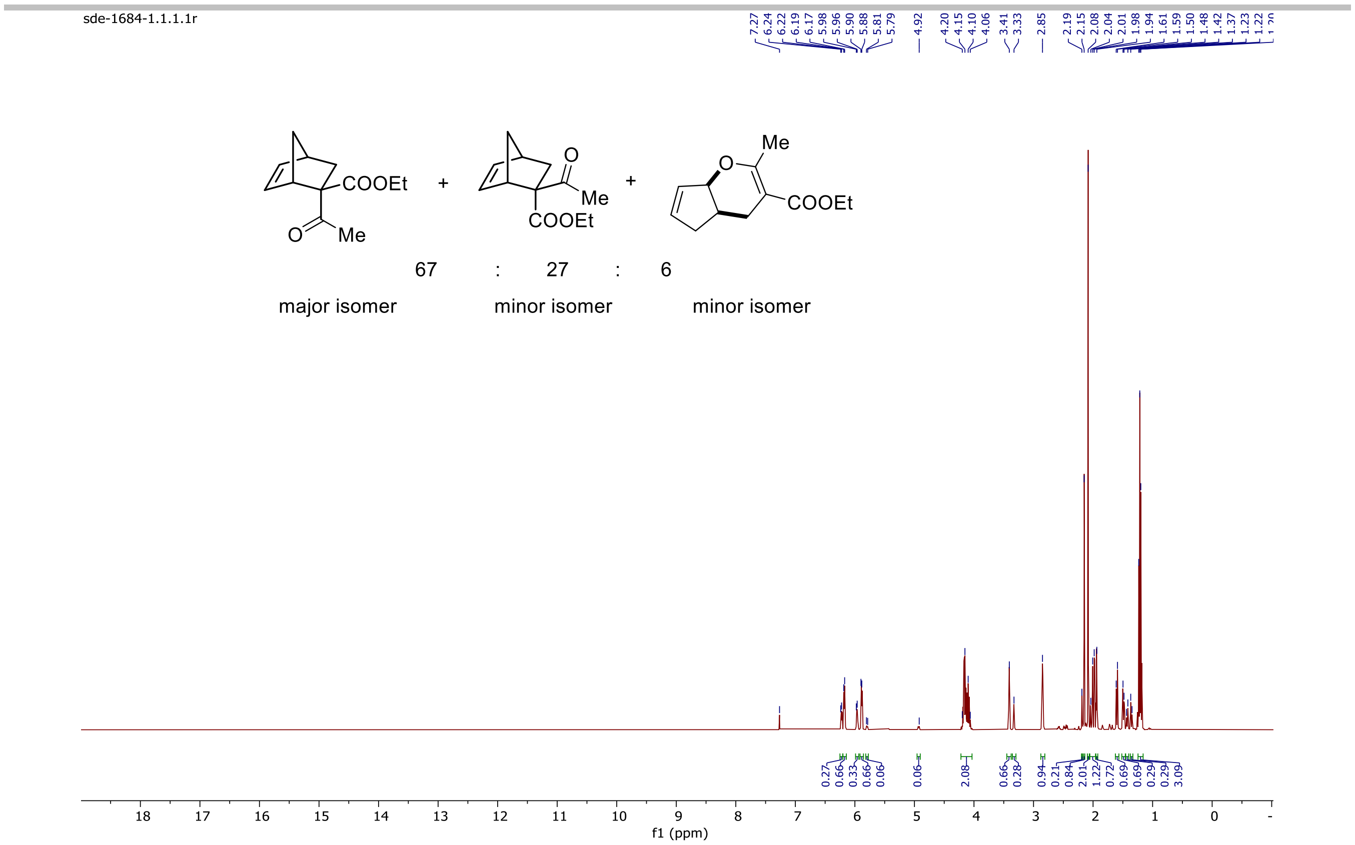

Figure S5. ^{1}H NMR spectra of the mixture of compounds **5a**, **5b** and **6.**

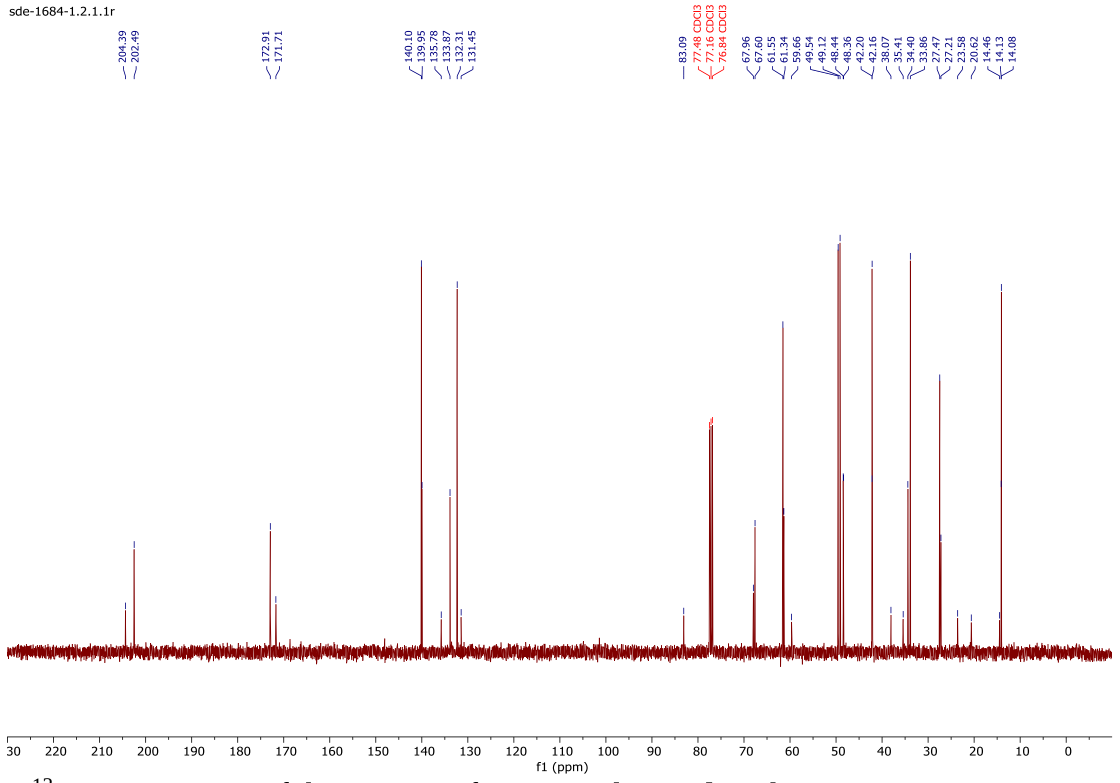

Figure S6. ^{13}C NMR spectra of the mixture of compounds **5a**, **5b** and **6.**

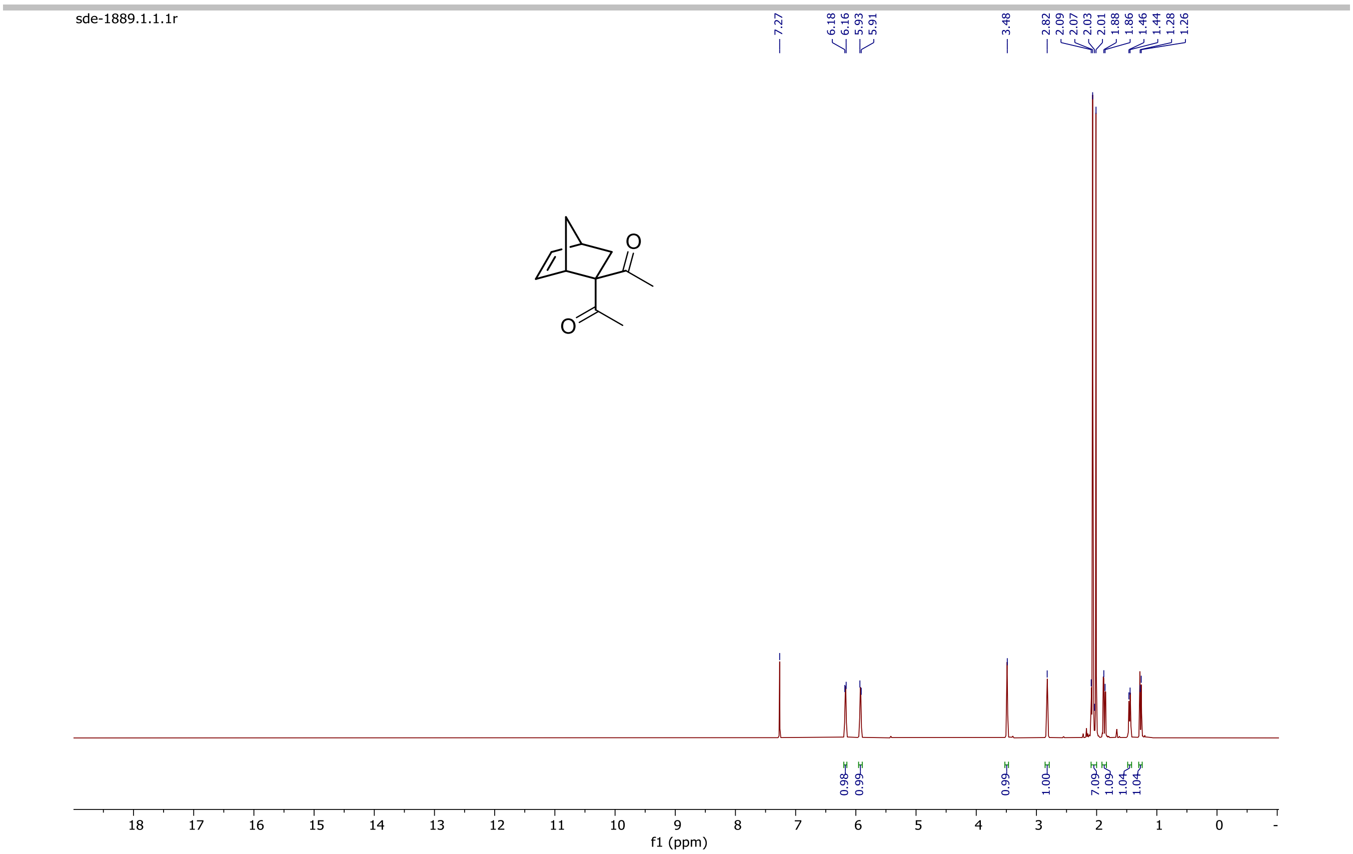

Figure S7. ^{1}H NMR spectra of compound **7.**

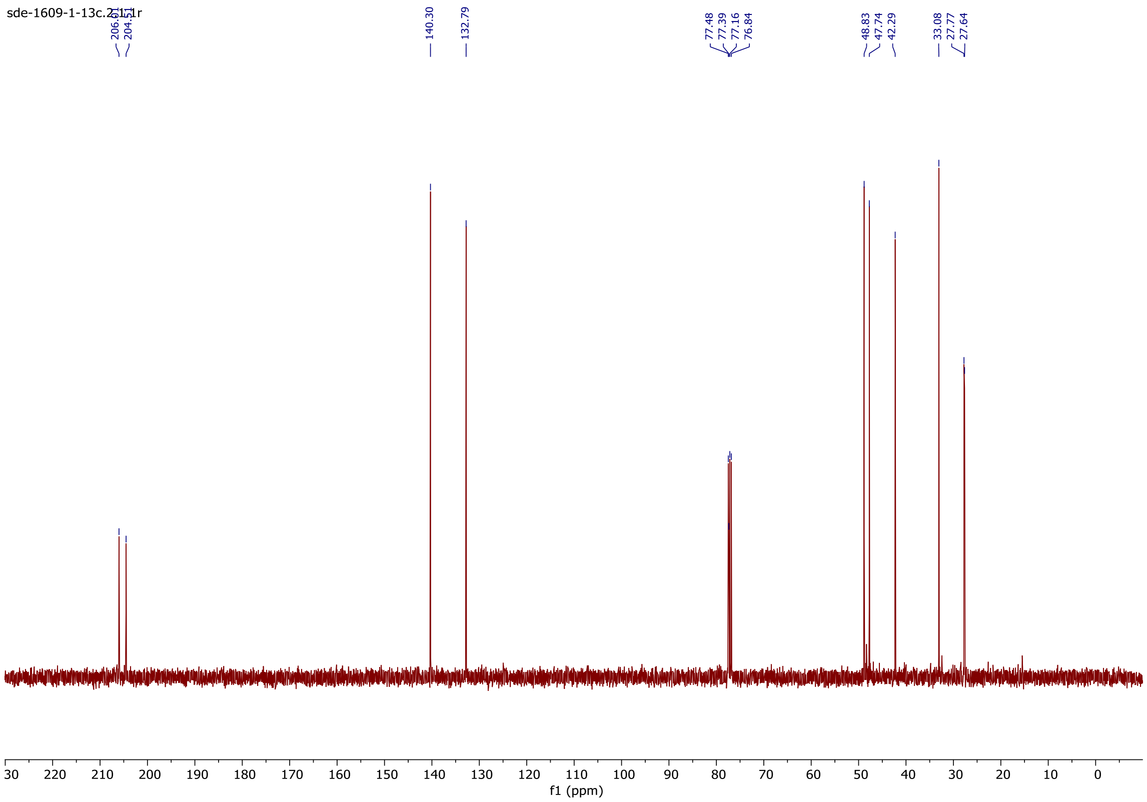

Figure S8. ^{13}C NMR spectra of compound **7.**

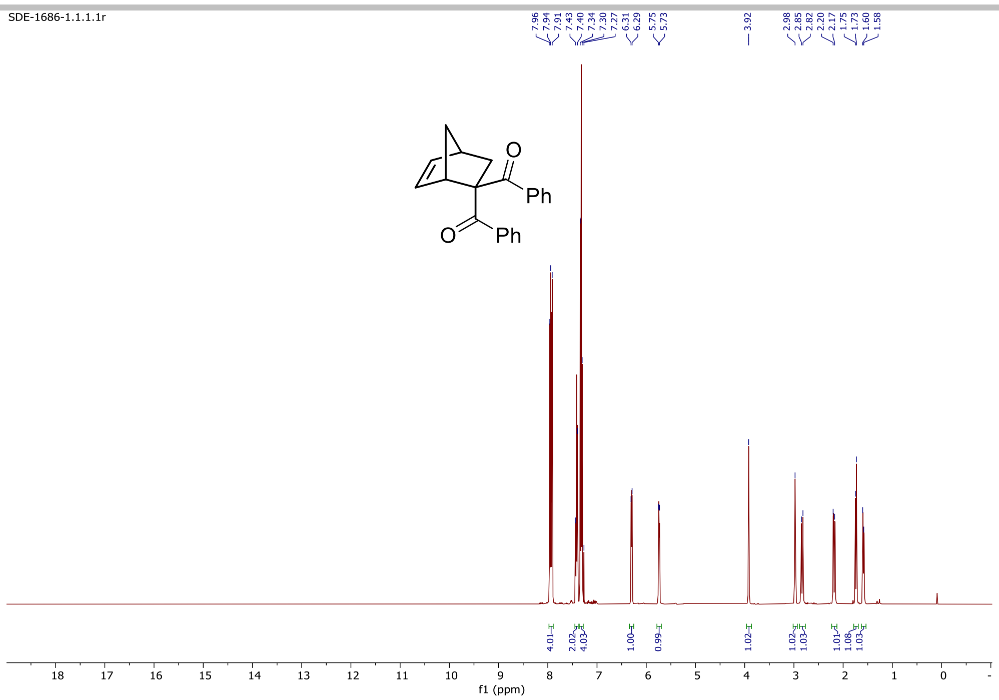

Figure S9. ^{1}H NMR spectra of compound **8.**

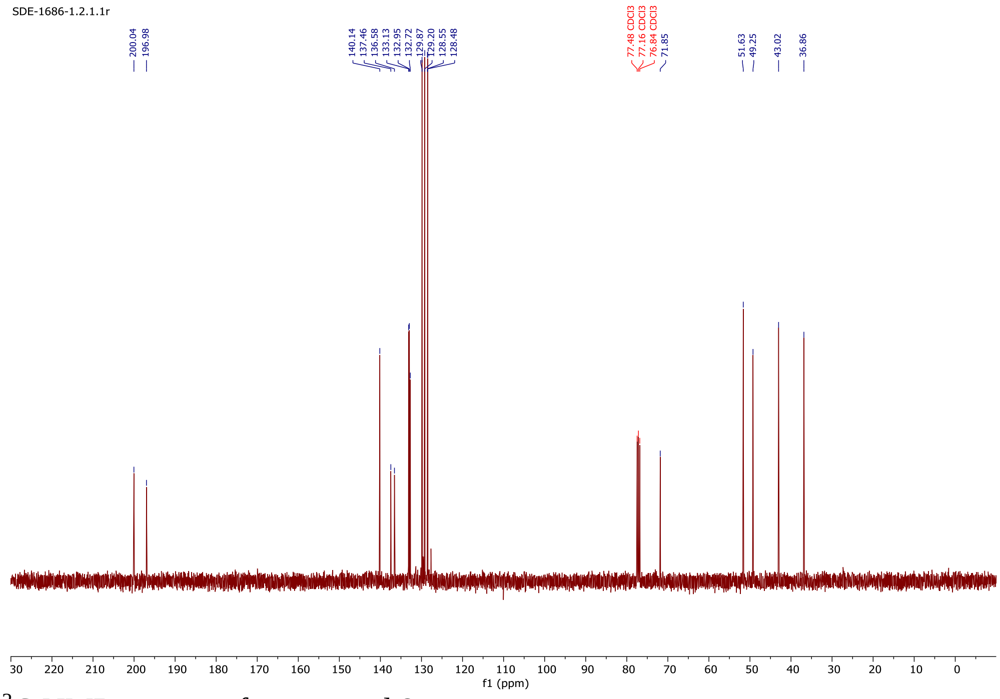

Figure S10. ^{13}C NMR spectra of compound **8.**

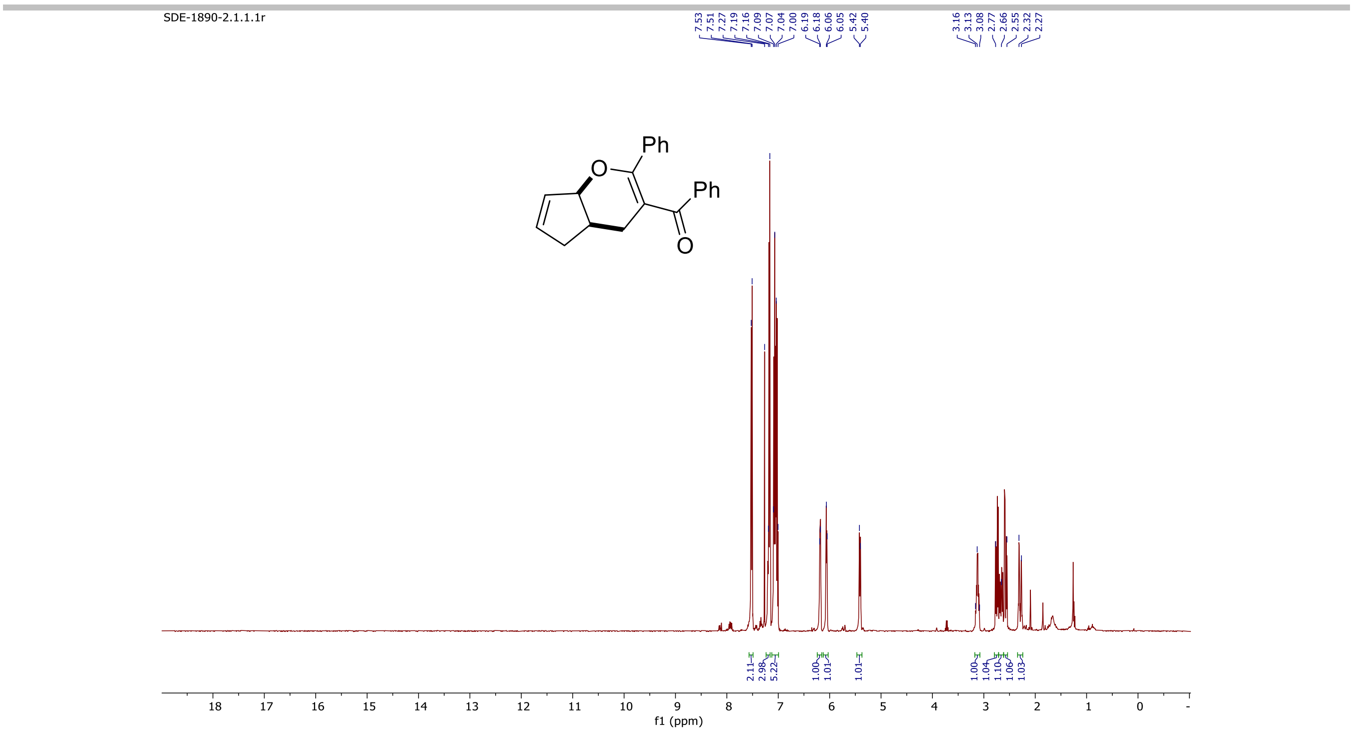

Figure S11. ^{1}H NMR spectra of compound **9.**

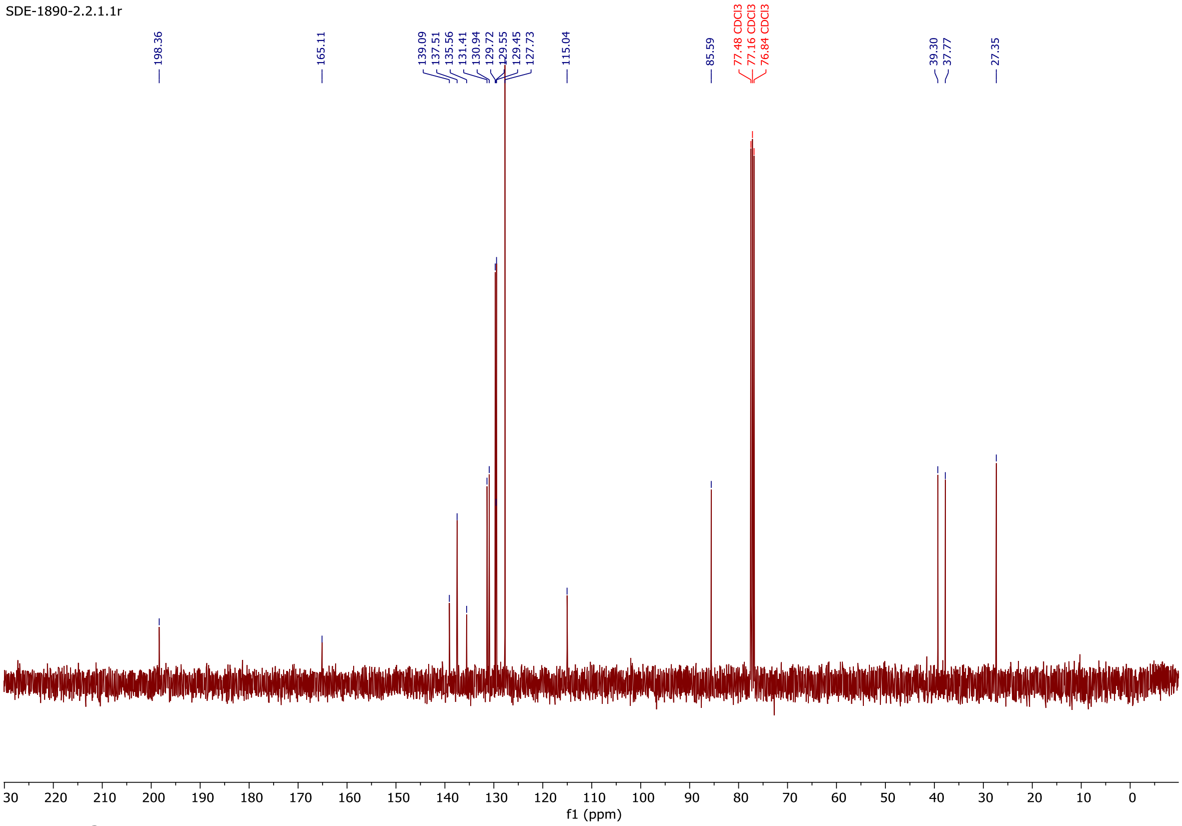

Figure S12. ^{13}C NMR spectra of compound **9.**

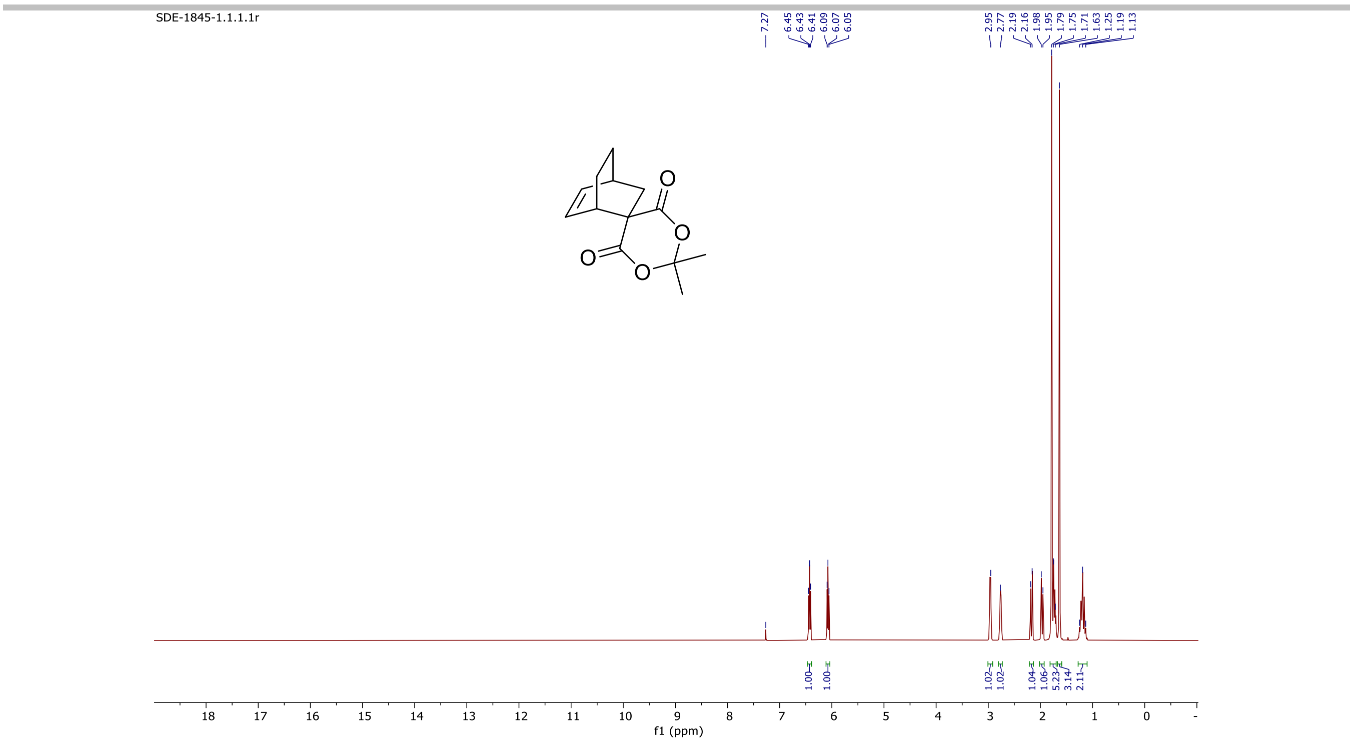

Figure S13. ^{1}H NMR spectra of compound **10.**

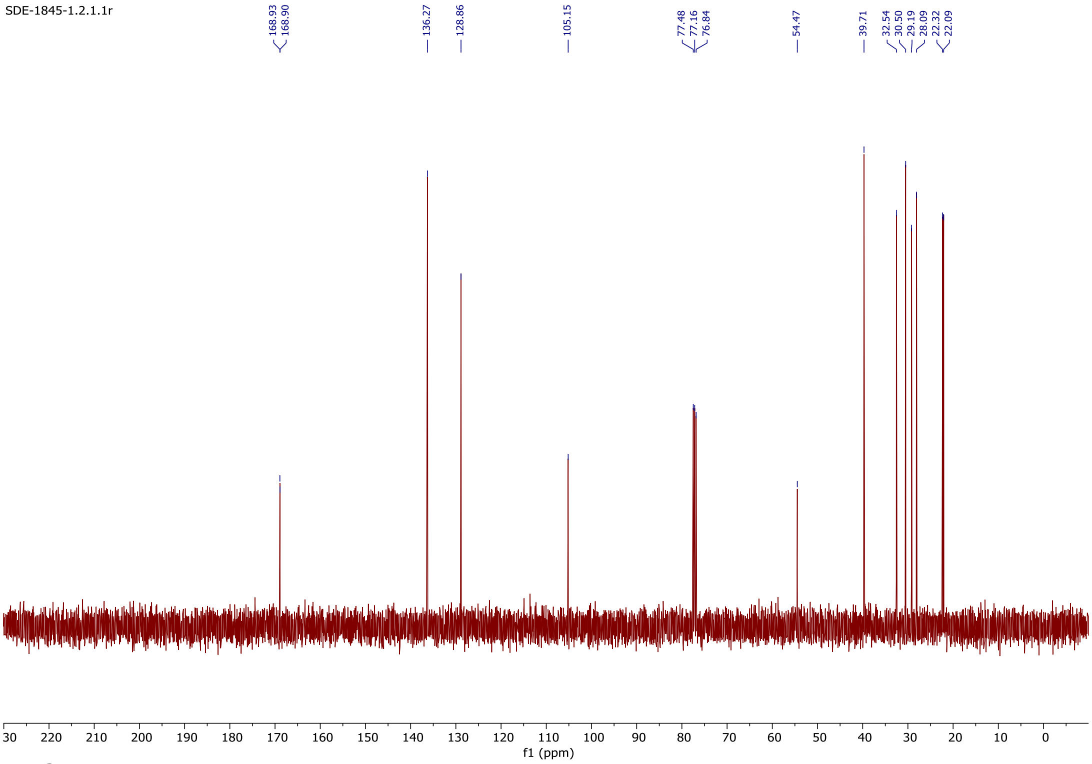

Figure S14. ^{13}C NMR spectra of compound **10.**

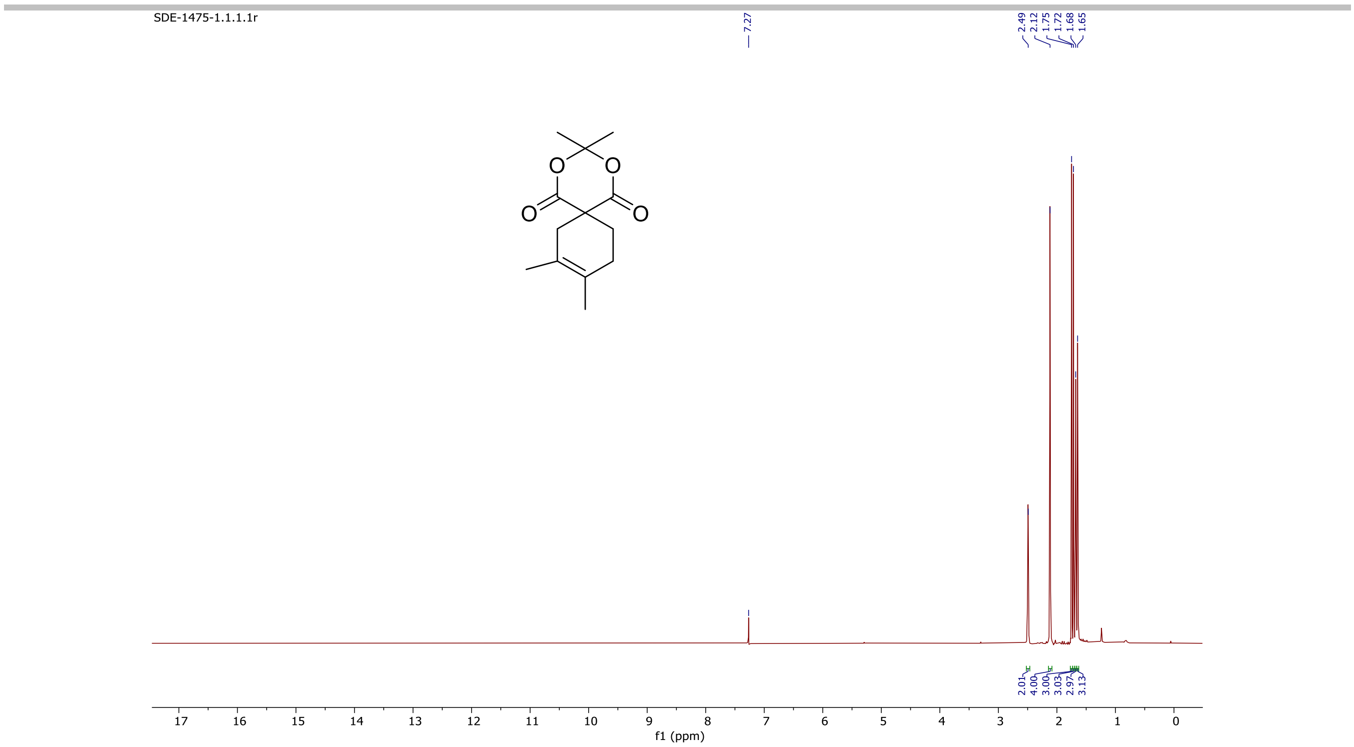

Figure S15. ^{1}H NMR spectra of compound **12.**

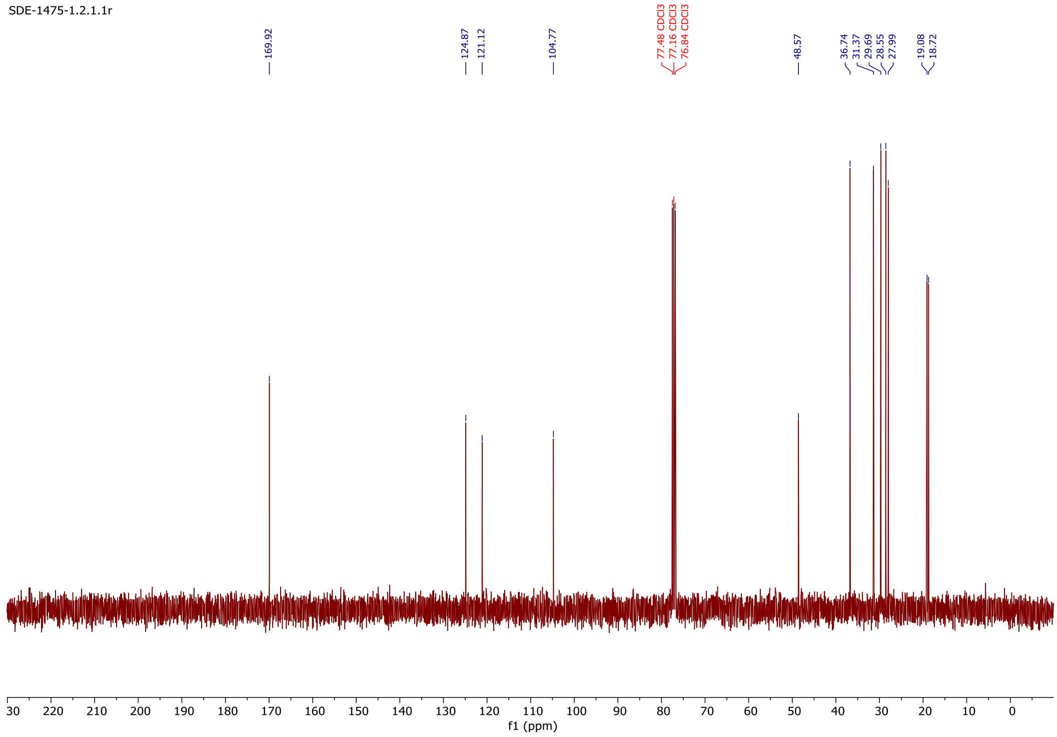

Figure S16. ^{13}C NMR spectra of compound **12.**

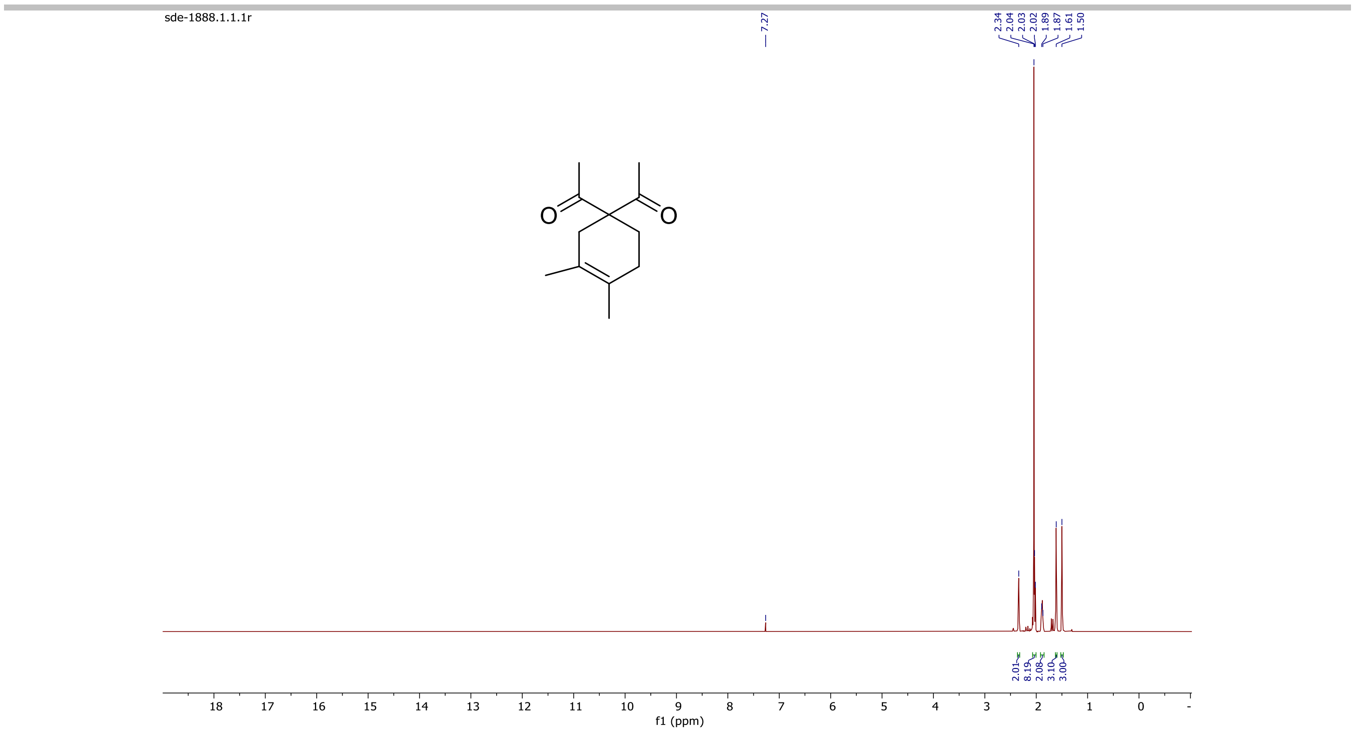

Figure S17. ^{1}H NMR spectra of compound **13.**

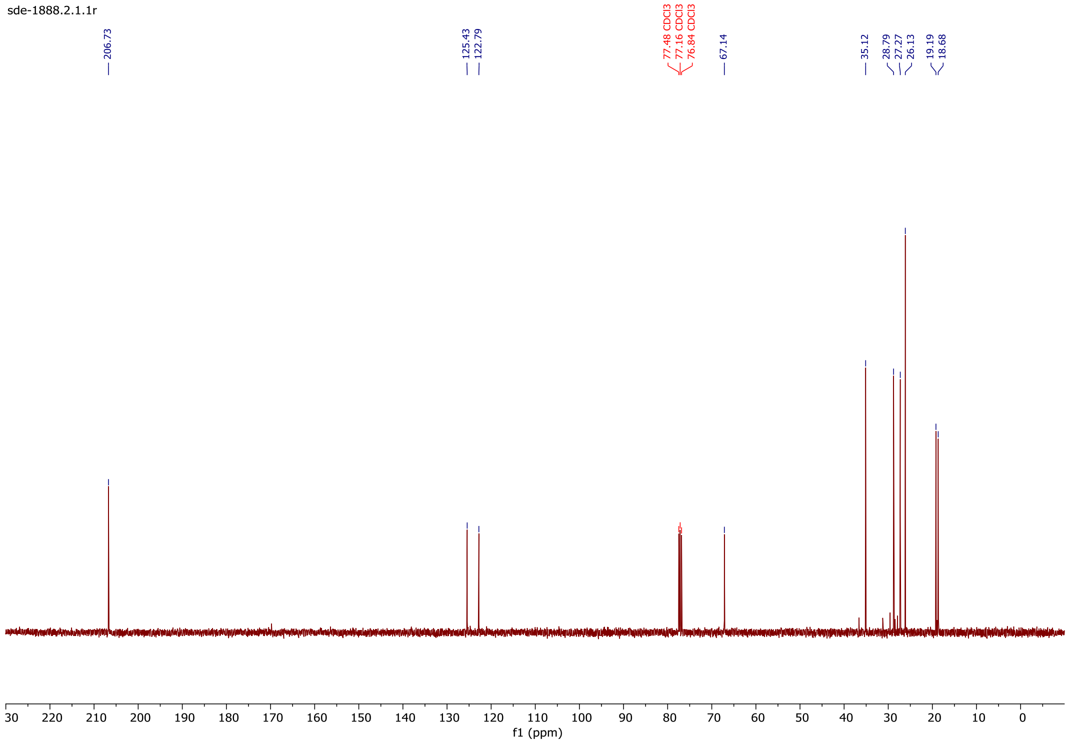

Figure S18. ^{13}C NMR spectra of compound **13.**

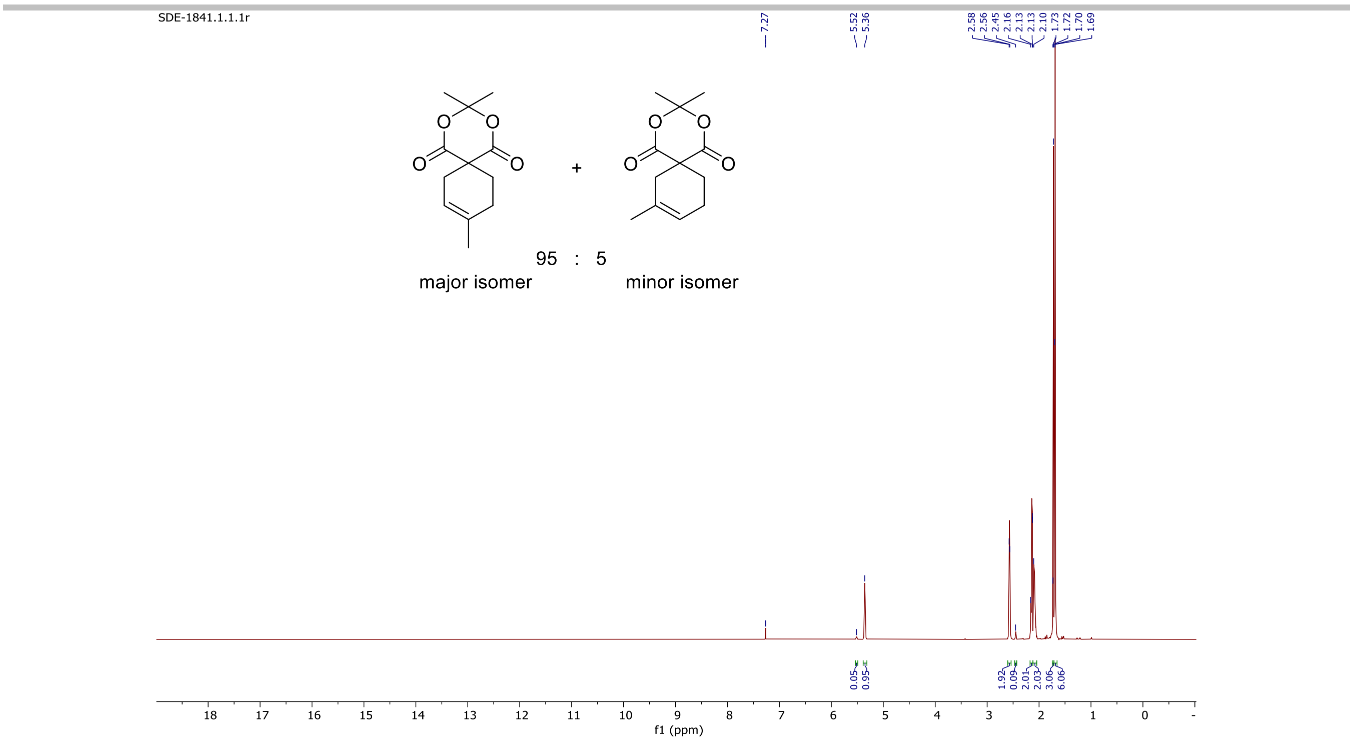

Figure S19. ^{1}H NMR spectra of the mixture of compounds **14a** and **14b.**

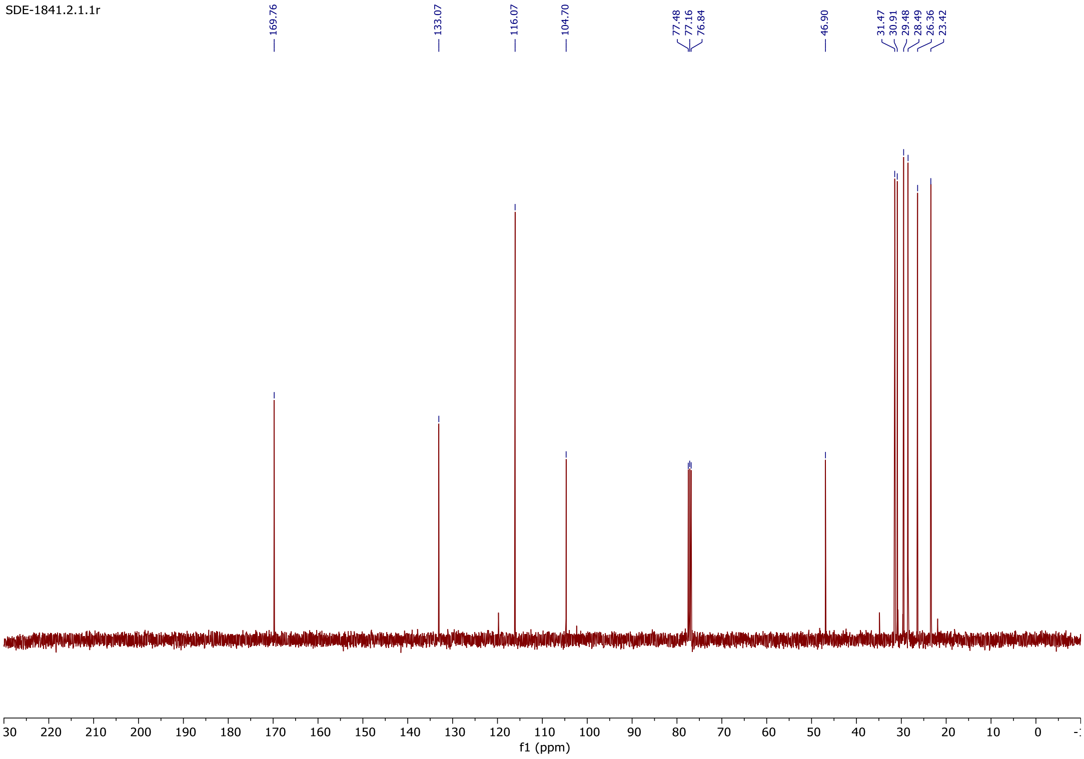

Figure S20. ^{13}C NMR spectra of the mixture of compounds **14a** and **14b.**

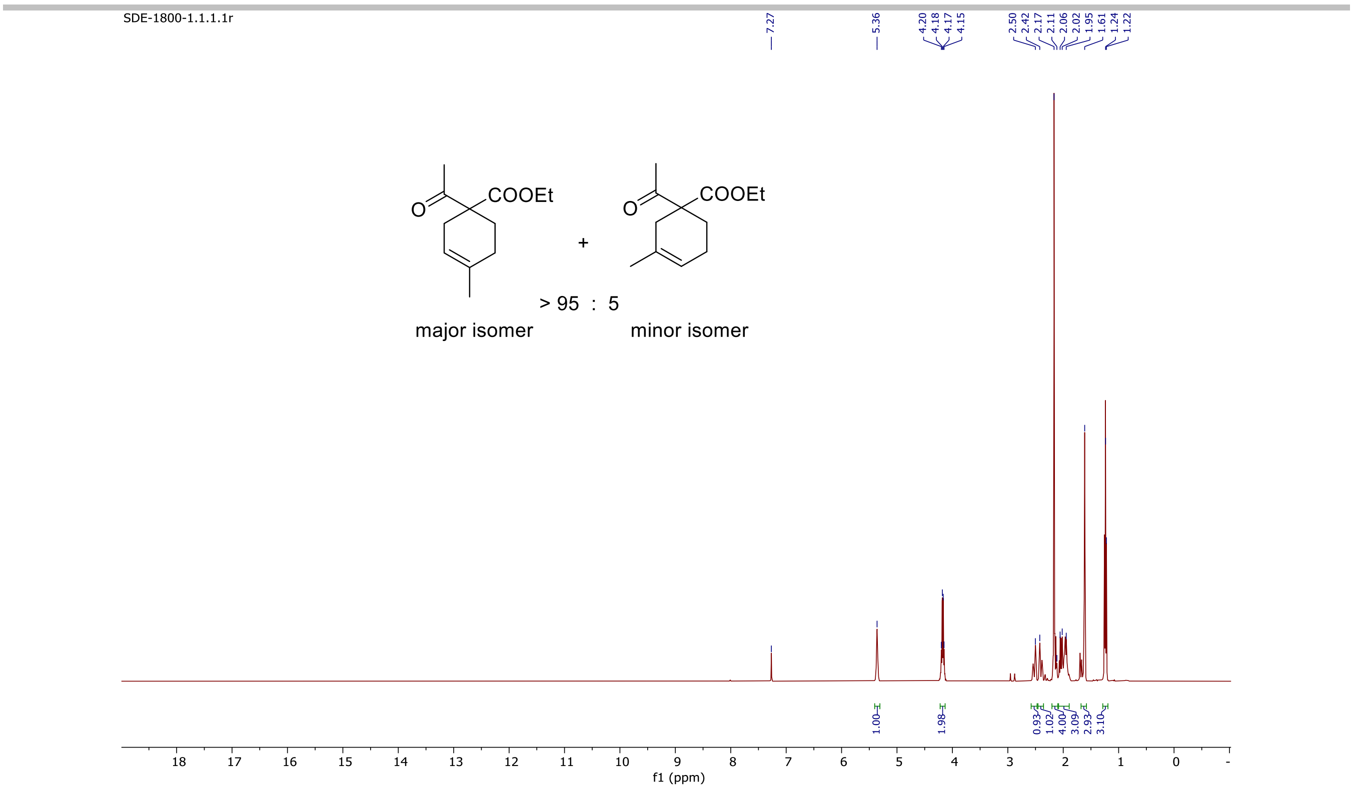

Figure S21. ^{1}H NMR spectra of the mixture of compounds **15a** and **15b.**

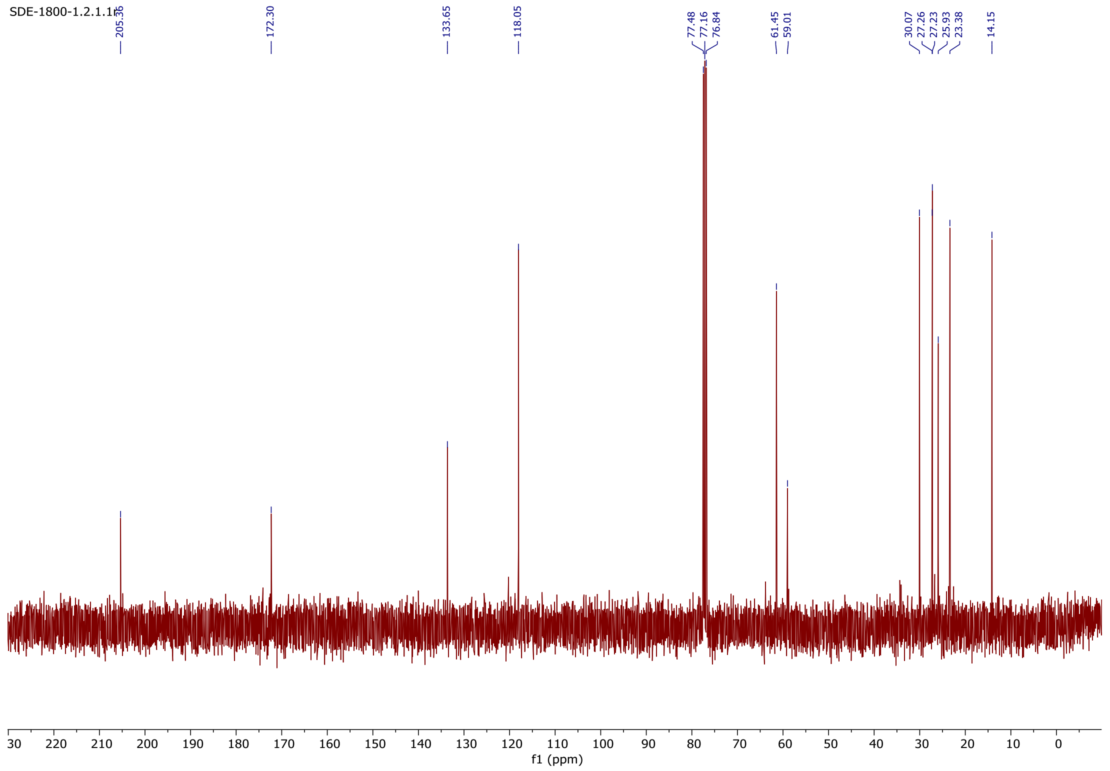

Figure S22. ^{13}C NMR spectra of the mixture of compounds **15a** and **15b.**

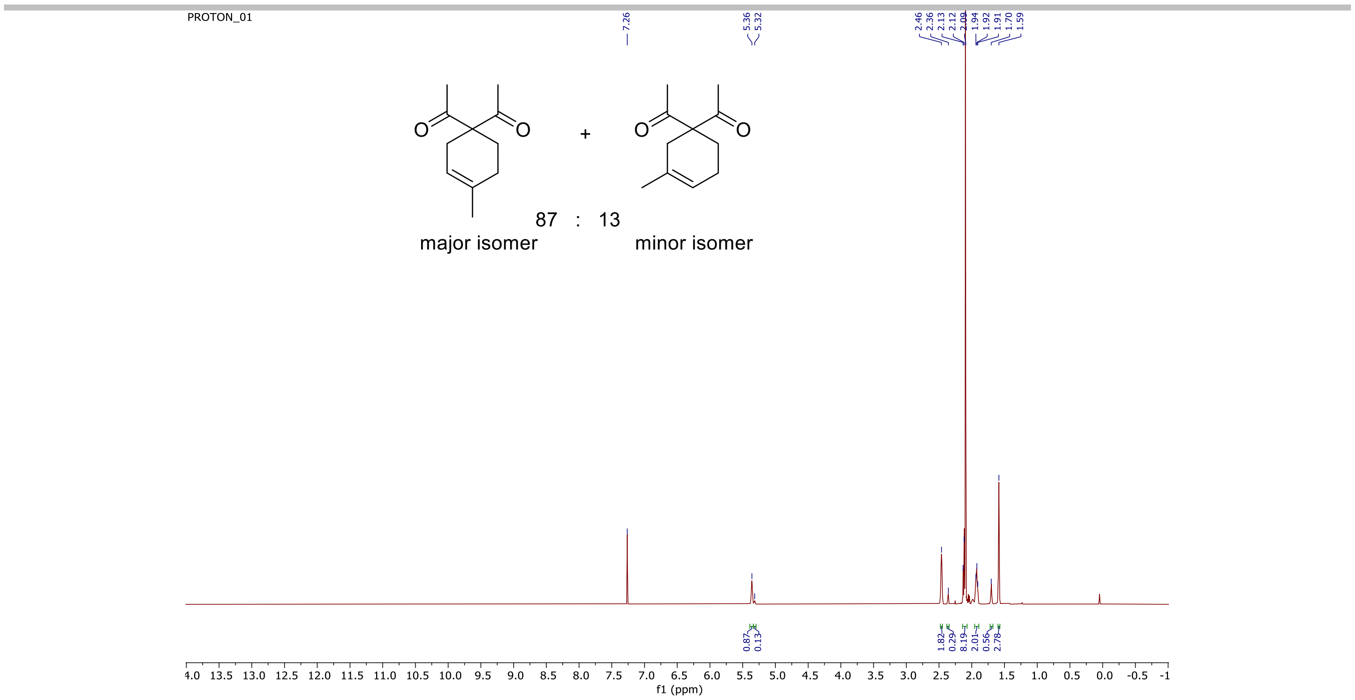

Figure S23. ^{1}H NMR spectra of the mixture of compounds **16a** and **16b.**

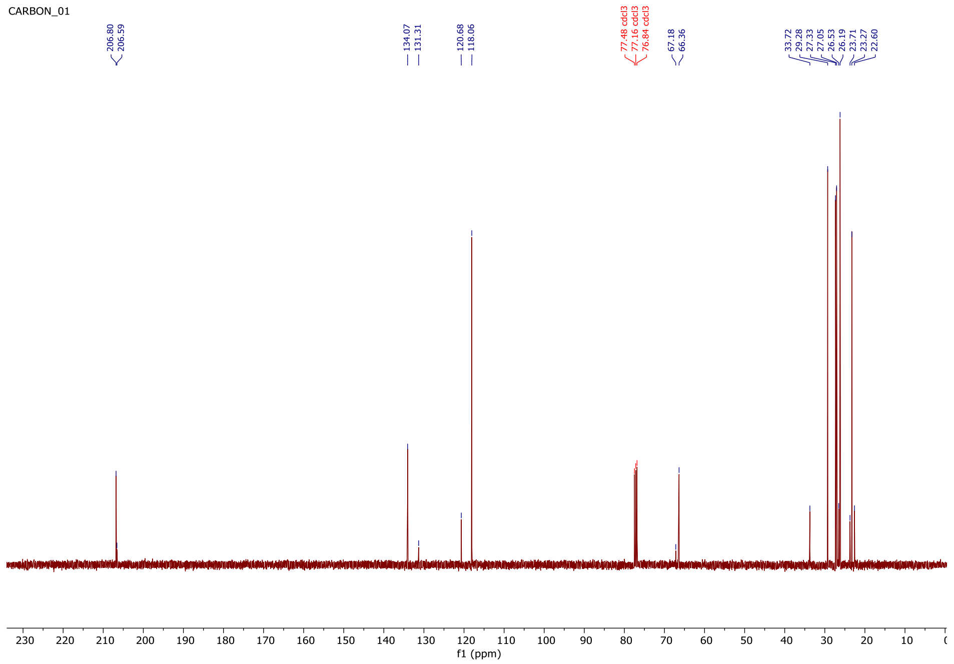

Figure S24. ^{13}C NMR spectra of the mixture of compounds **16a** and **16b.**

3. ^{1}H NMR spectra of reaction mixtures formed during bromination of compounds 4 and 5

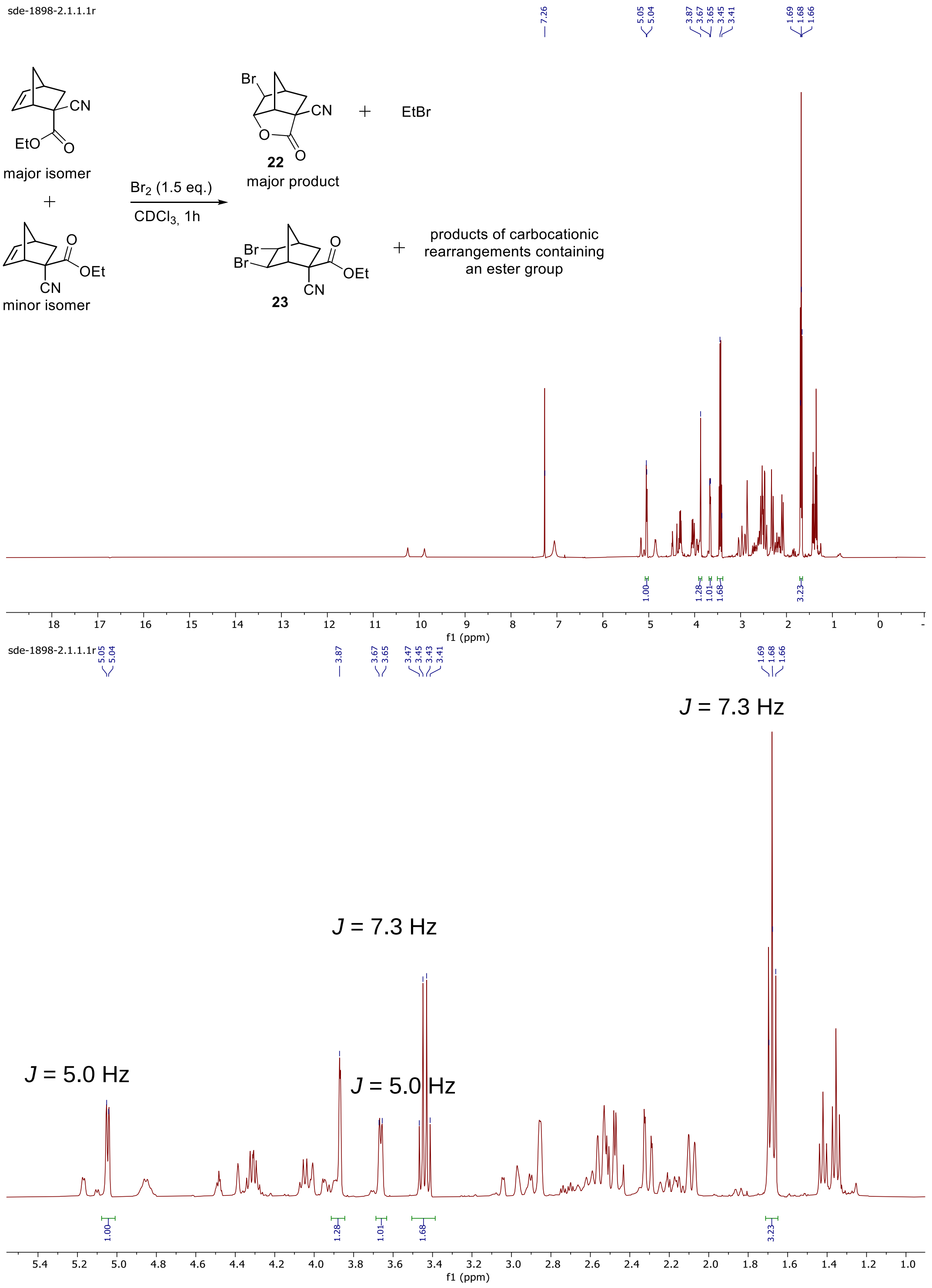

Figure S25. ^{1}H NMR spectrum of the reaction mixture of bromination of compounds **4a** and **4b**. The characteristic signals of the main reaction products, as well as EtBr, are labeled.

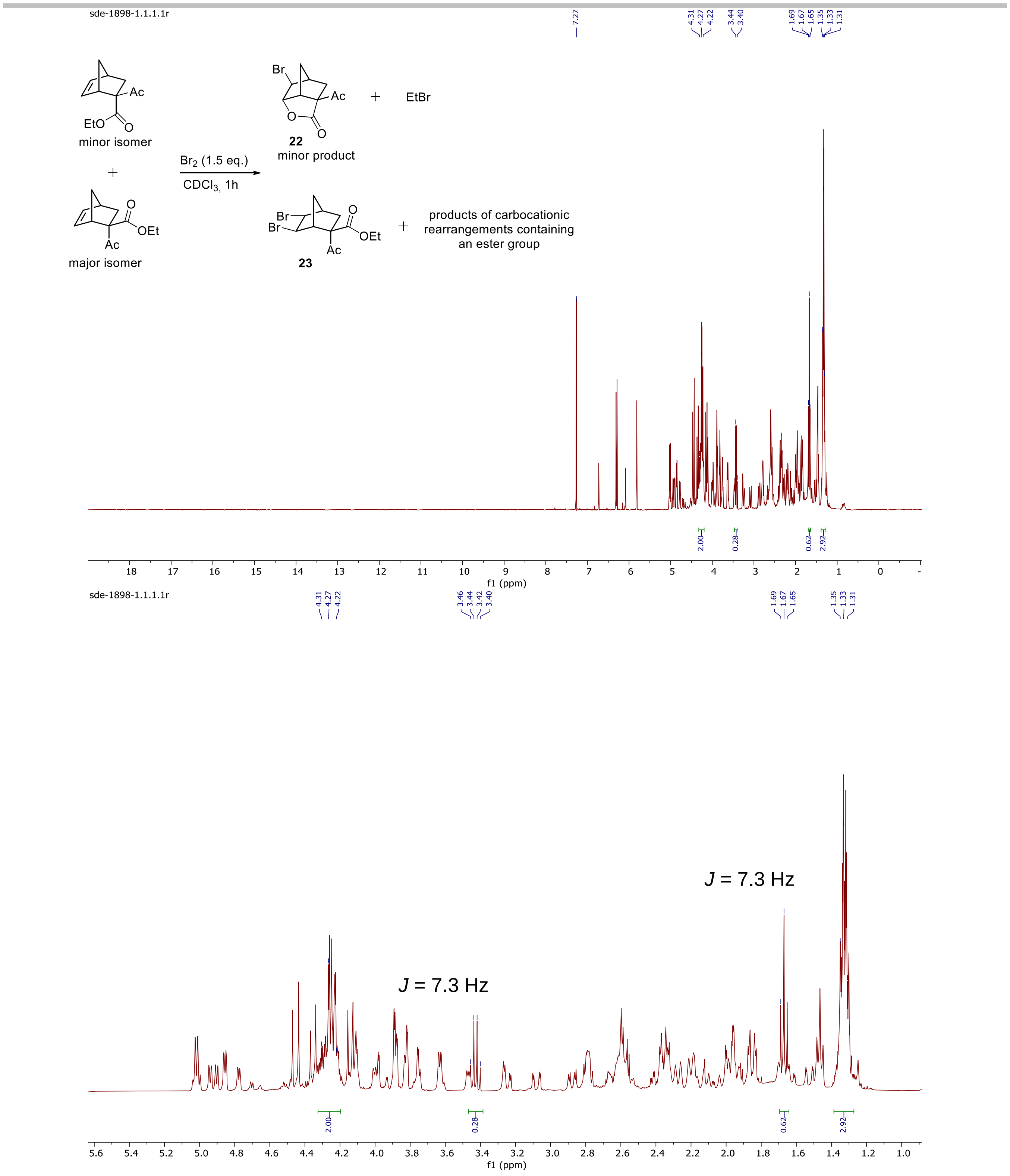

Figure S26. ^{1}H NMR spectrum of the reaction mixture of bromination of compounds **5a** and **5b**. The characteristic signals of the main reaction products, as well as EtBr, are labeled.

4. LC–MS/MS spectra of compound mixtures

HPLC-MS/MS method parameters for compound analysis

Agilent 1290 Infinity II – 5500 QTRAP (Sciex)				
**Chromatographic parameters**				
Solvent A	0,1% HCOOH in H_2O			
Solvent B	0,1% HCOOH in CH_3CN			
Chromatographic column	Eclipse XDB C18, 150*3 mm, 5 µm (Agilent)			
Gradient	Time, min	Flow rate, mL/min	%A	%B
	0,00	0,4	80,0	80,0
	1,00	0,4	80,0	80,0
	10,0	0,4	20,0	20,0
	12,0	0,4	20,0	20,0
	12,1	0,4	80,0	80,0
	15,0	0,4	80,0	80,0
Column temperature	40ºC			
Autosampler temperature	8ºC			
Injection volume	10 µL			
Total analysis time	15 min			
**MS/MS parameters**				
Ion source type	Turbo Spray			
Ionization mode	Positive/Negative			
Source temperature, ºC	630			
Nebulizer voltage (IS), V	5500			
Curtain gas (CUR), psi	30			
Nebulizer gas (GS1), psi	70			
Heater gas (GS2), psi	70			
Mass range	100 - 1000			
Scanning quadrupole	Q3			
Declustering potential, V	60			

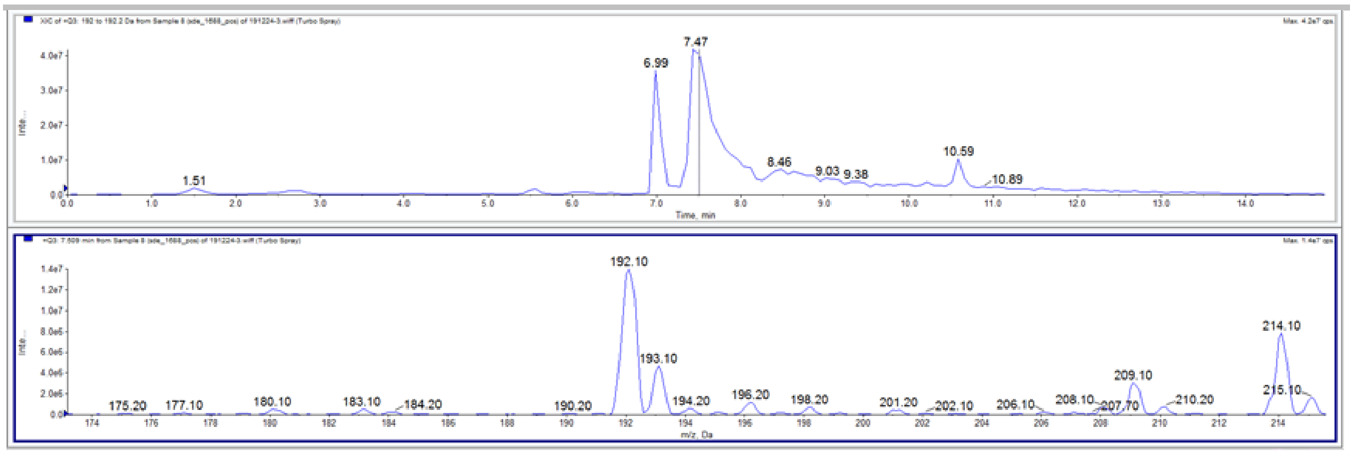

Figure S27. LC-MS/MS spectra of the compounds **4a** and **4b** mixture.

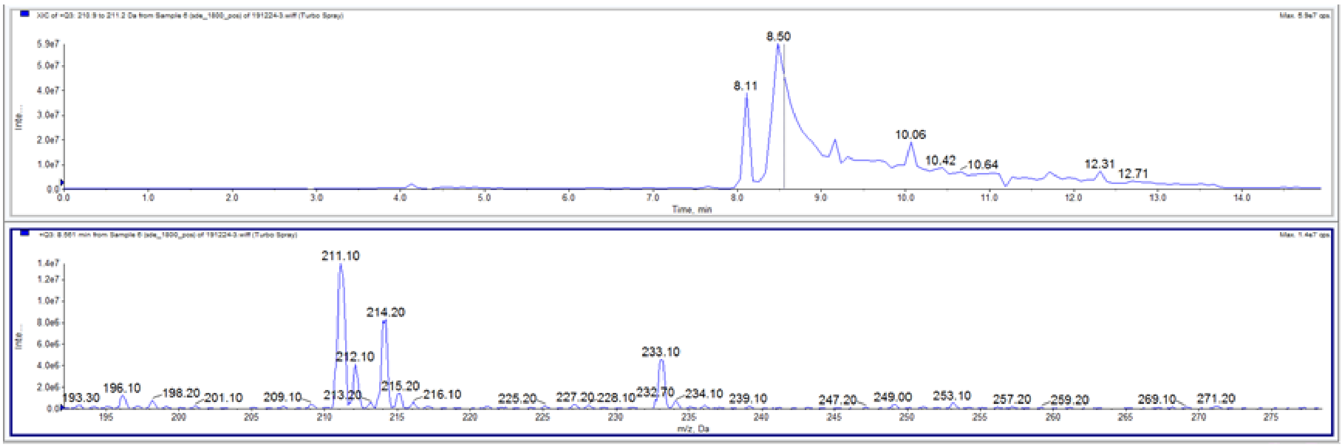

Figure S28. LC-MS/MS spectra of the compounds **5a**, **5b** and **6** mixture.

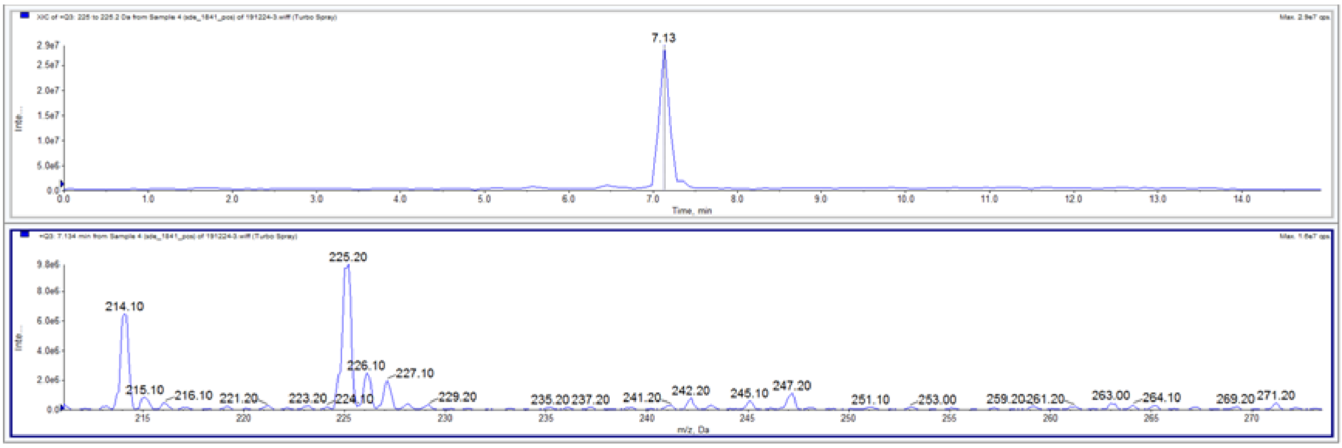

Figure S29. LC-MS/MS spectra of the compounds **14a** and **14b** mixture.

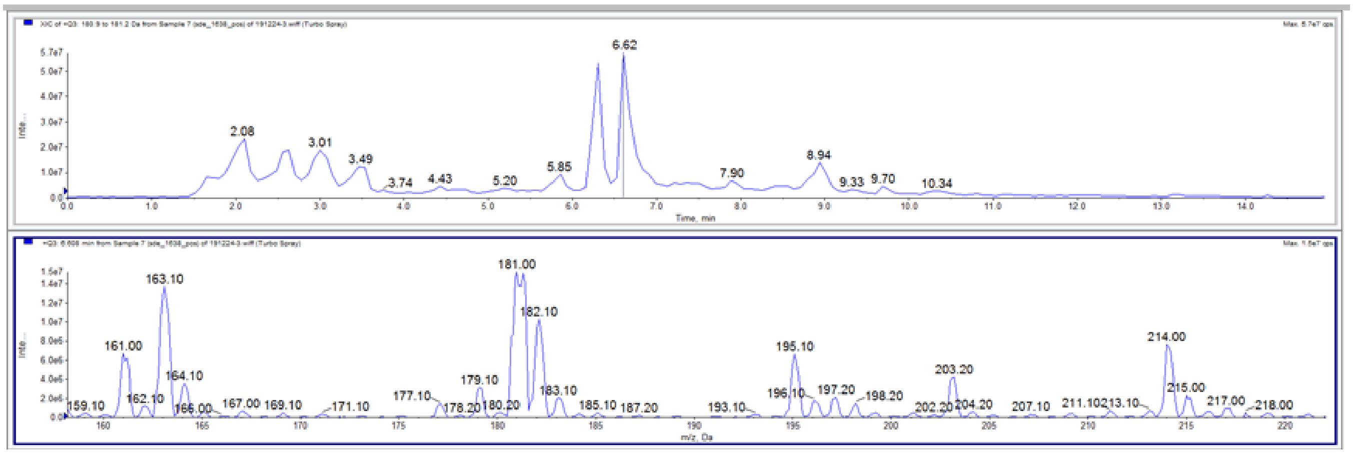

Figure S30. LC-MS/MS spectra of the compounds **16a** and **1b** mixture.

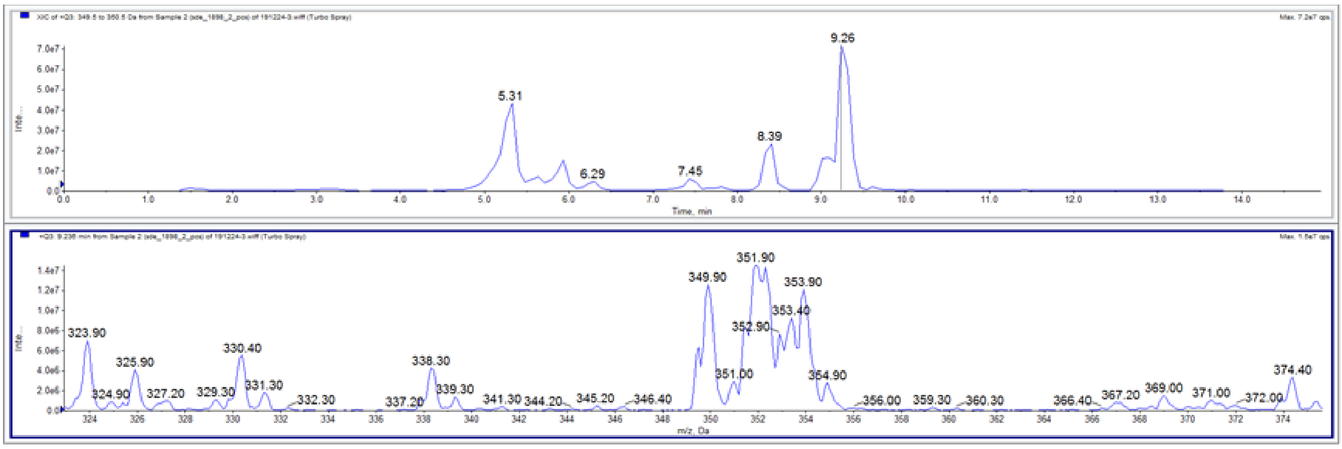

Figure S31. LC-MS/MS spectra of the the reaction mixture **4a** + **4b** + Br_2.

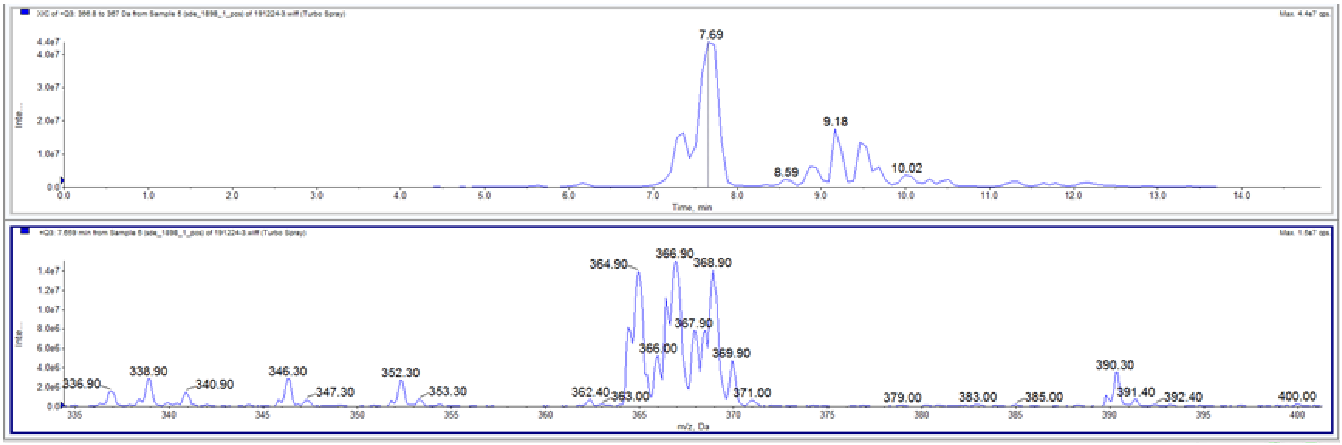

Figure S32. LC-MS/MS spectra of the the reaction mixture **5a** + **5b** + Br_2.

References

1. Yuan, X.; Yang, J.; Yang, X.; He, N.; Han, M.; Lin, J.; Yu, W.; Cheng, X.; Jin, Y. Methylene-Bridged Dimerization by Cu-Catalyzed Deconstructive C-C Cleavage of Oxacycloalkane. *Org. Lett.* **2023**, *25*, 5630–5635, doi:10.1021/acs.orglett.3c02015.
2. Mellor, J.M.; Webb, C.F. Stereochemistry of the Diels-Alder Reaction: Steric Effects of the Dienophile on Endo-Selectivity. *J. Chem. Soc. Perkin Trans. 2* **1974**, 17–22, doi:10.1039/p29740000017.
3. Yamauchi, M.; Watanabe, T. High Asymmetric Induction in the Diels-Alder Reactions of 1-Benzoyl-1-Phenyl- Menthyloxycarbonylethene. *J. Chem. Soc. Chem. Commun.* **1988**, 27–28.
4. Snider, B.B.; Smith, R.B. Mn(III)-Based Oxidative Free-Radical Cyclizations of Alkenyl Meldrum's Acids. *Tetrahedron* **2002**, *58*, 25–34, doi:10.1016/S0040-4020(01)01054-7.
5. Zia-Ebrahimi, M.; Huffman, G.W. Synthesis and Utility of a Novel Methylene Meldrum's Acid Precursor. *Synthesis (Stuttg).* **1996**, 215–218, doi:10.1055/s-1996-4201.
6. Burke, D.J.; Kawauchi, T.; Kade, M.J.; Leibfarth, F.A.; McDearmon, B.; Wolffs, M.; Kierstead, P.H.; Moon, B.; Hawker, C.J. Ketene-Based Route to Rigid Cyclobutanediol Monomers for the Replacement of BPA in High Performance Polyesters. *ACS Macro Lett.* **2012**, *1*, 1228–1232, doi:10.1021/mz300497m.
